# *Pectobacterium atrosepticum* and *Pectobacterium carotovorum* Harbor Distinct, Independently Acquired Integrative and Conjugative Elements Encoding Coronafacic Acid that Enhance Virulence on Potato Stems

**DOI:** 10.3389/fmicb.2016.00397

**Published:** 2016-03-31

**Authors:** Preetinanda Panda, Bhanupratap R. Vanga, Ashley Lu, Mark Fiers, Peter C. Fineran, Ruth Butler, Karen Armstrong, Clive W. Ronson, Andrew R. Pitman

**Affiliations:** ^1^The Bio-Protection Research CentreLincoln, New Zealand; ^2^Plant Pathology, The New Zealand Institute for Plant and Food Research LimitedLincoln, New Zealand; ^3^Department of Microbiology and Immunology, University of OtagoDunedin, New Zealand

**Keywords:** soft rot enterobacteriaceae, pathogenicity island, blackleg, CRISPR-Cas chromosomal editing, horizontal gene transfer

## Abstract

Integrative and conjugative elements (ICEs) play a central role in the evolution of bacterial virulence, their transmission between bacteria often leading to the acquisition of virulence factors that alter host range or aggressiveness. Much is known about the functions of the virulence determinants that ICEs harbor, but little is understood about the cryptic effects of ICEs on their host cell. In this study, the importance of horizontally acquired island 2 (HAI2), an ICE in the genome of *Pectobacterium atrosepticum* SCRI1043, was studied using a strain in which the entire ICE had been removed by CRISPR-Cas-mediated genome editing. HAI2 encodes coronafacic acid, a virulence factor that enhances blackleg disease of potato stems caused by *P. atrosepticum* SCRI1043. As expected, deletion of HAI2 resulted in reduced blackleg symptoms in potato stems. A subsequent screen for HAI2-related ICEs in other strains of the *Pectobacterium* genus revealed their ubiquitous nature in *P. atrosepticum*. Yet, HAI2-related ICEs were only detected in the genomes of a few *P. carotovorum* strains. These strains were notable as blackleg causing strains belonging to two different subspecies of *P. carotovorum*. Sequence analysis of the ICEs in different strains of both *P. atrosepticum* and *P. carotovorum* confirmed that they were diverse and were present in different locations on the genomes of their bacterial host, suggesting that the *cfa* cluster was probably acquired independently on a number of occasions via chromosomal insertion of related ICEs. Excision assays also demonstrated that the ICEs in both *P. atrosepticum* and *P. carotovorum* are mobilized from the host chromosome. Thus, the future spread of these ICEs via lateral gene transfer might contribute to an increase in the prevalence of blackleg-causing strains of *P. carotovorum*.

## Introduction

Historically, the enterobacterial phytopathogen *Pectobacterium atrosepticum* was considered the primary pathogen responsible for the rotting or wilting of stems on growing potato plants, referred to as blackleg (Pérombelon, 2002). Recent surveys to identify the source of blackleg symptoms in the field, however, led to the isolation of numerous species and subspecies of *Pectobacterium* from disease lesions. For example, *P*. *carotovorum* subsp. *brasiliensis* was obtained from potatoes in Brazil, where it was the major cause of blackleg (Duarte et al., 2004). *P. carotovorum* subsp. *brasiliensis* was subsequently detected in potato cropping systems in Israel (Ma et al., 2007), South Africa (van der Merwe et al., 2010), Canada (De Boer et al., 2012), New Zealand (Panda et al., 2012), Zimbabwe (Ngadze et al., 2012) and in the Netherlands (Leite et al., 2014), suggesting the pathogen has a global impact on potato. *P. wasabiae* was also collected from potato plants with blackleg symptoms (Pérombelon and Kelman, 1980; De Boer, 2002). Isolates of *P. wasabiae* were initially thought to be secondary invaders, until their vacuum infiltration into tubers was shown to induce blackleg (de Haan et al., 2008). *P. wasabiae* was subsequently detected in a multitude of potato growing regions including the United States, New Zealand, Iran, and South Africa (Kim et al., 2009; Pitman et al., 2010; Baghaee-Ravari et al., 2011; Moleleki et al., 2013).

The isolation of divergent *Pectobacterium* strains with the capacity to cause blackleg suggests that they may share common virulence determinants associated with stem infection. The genome sequence of *P. atrosepticum* SCRI1043 (Bell et al., 2004) identified various loci associated with virulence, including a coronafacic acid (Cfa) biosynthetic gene cluster. Cfa is a component of the toxin coronatine (Cor), an important virulence factor for some pathovars of the bacterial pathogen *Pseudomonas syringae* (Bender et al., 1999). Cor is formed by the conjugation of the Cfa polyketide to coronamic acid (Cma), an ethylcyclopropyl amino acid derived from isoleucine (Parry et al., 1994). Unlike *P. syringae, P. atrosepticum* SCRI1043 does not encode genes for the production of Cma, suggesting that it produces an alternative polyketide phytotoxin, or that Cfa acts alone during disease development. In *P. syringae*, Cor mimics jasmonate 12-oxo-phytodienoic acid. Jasmonate 12-oxo-phytodienoic acid is a precursor to jasmonic acid (Weiler et al., 1994). Cor acts by stimulating jasmonate response pathways (Zhao et al., 2003) and suppressing salicylic acid-mediated defenses (Uppalapati et al., 2007). The function of Cfa in *P. atrosepticum* is unknown, but a transposon insertion in the *cfa* cluster reduced the capacity of the pathogen to cause blackleg symptoms on potato (Bell et al., 2004). Thus, Cfa probably enables the pathogen to manipulate host immunity during blackleg-related infection in potato.

In *P. atrosepticum* SCRI1043, the *cfa* biosynthetic cluster is harbored on a putative horizontally acquired island, HAI2 (Bell et al., 2004). HAI2 is 97,875 bp in size, has a G+C content of 48.30% compared with 50.97% for the entire genome and has 99 predicted coding DNA sequences (CDSs; Vanga et al., 2012). It shows strong similarity to a family of integrative and conjugative elements (ICEs) that include SPI-7 from *Salmonella enterica* serovar Typhi TY2 (Bueno et al., 2004), PAP1 from *P. aeruginosa* PA14 (He et al., 2004), and PPHGI-1 from *P. syringae* pv. *phaseolicola* 1302A (Pitman et al., 2005).

Integrative and conjugative elements are typically stably integrated into the chromosome, flanked by direct repeats and inserted at the 3′ end of a tRNA gene (Hacker and Kaper, 2000). They can also excise from the chromosome, resulting in the formation of an extrachromosomal circular form that facilitates transfer between donor and recipient cells. HAI2 is no exception, integrated within the genome of *P. atrosepticum* SCRI1043 at a bacterial attachment site (*attB^*0515*^*) located within the *phe*-tRNA gene immediately downstream of *ECA0515* (Vanga et al., 2012). HAI2 can excise at low frequency from the chromosome (Vanga et al., 2012), a process that is induced *in planta* (Vanga et al., 2015). Mobilization and transfer of ICEs plays a central role in the evolution of pathogens by transferring large amounts of genetic information between bacteria (Morschhäuser et al., 2000). As the *cfa* biosynthetic cluster has only been detected in strains of *P. atrosepticum* and *P. carotovorum* responsible for blackleg (Bell et al., 2004; Slawiak and Lojkowska, 2009; Panda et al., 2015), it is possible that independent acquisition of HAI2 has contributed to the capacity of both species to cause the disease by transferring this important virulence factor.

In this study, we examined the role of HAI2 in aggressiveness of *P. atrosepticum* SCRI1043 on potato stems by conducting pathogenicity tests using SCRI1043ΔHAI2, an ‘ICE-less’ strain previously generated using endogenous type I-F CRISPR-Cas chromosomal targeting (Vercoe et al., 2013; Dy et al., 2014; Richter et al., 2014). CRISPR-Cas systems include clustered regularly interspaced short palindromic repeats (CRISPRs) and their associated Cas proteins, and are an adaptive immune system in bacteria against foreign genetic elements (Dy et al., 2013). We also studied the distribution and genetic organization of the *cfa* biosynthetic clusters in a variety of related strains of the *Pectobacterium* genus from potato to understand if independent acquisition of HAI2 may have led to the capacity of different species of *Pectobacterium* to cause blackleg.

## Materials and Methods

### Bacterial Strains and DNA Manipulations

SCRI1043ΔHAI2 was used to study the function of HAI2. SCRI1043ΔHAI2 is a derivative of *P. atrosepticum* SCRI1043 that has the entire ICE removed from the genome using CRISPR-Cas-mediated genome targeting (Vercoe et al., 2013) followed by curing the strain of the chromosomal-targeting crRNA expression plasmid (Richter et al., 2014). An additional 87 strains of the *Pectobacterium* genus isolated from potato in New Zealand (Pitman et al., 2008) and a variety of type strains and genetically or biochemically characterized isolates obtained from the International Collection of Micro-organisms from Plants (ICMP), Landcare Research, New Zealand (unless otherwise stated; Supplementary Table S1) were also used to examine the distribution of HAI2 and other closely related ICEs. All bacteria were routinely grown in Lysogeny Broth medium (LB) or Minimal Medium (MM) at 28°C for 24–48 h. Genomic DNA was extracted from liquid cultures using the DNeasy tissue kit (Qiagen) following the manufacturer’s instructions.

### Pathogenicity Tests on Potato

The virulence of SCRI1043ΔHAI2 was compared with wild type *P. atrosepticum* SCRI1043 by carrying out blackleg assays on potato stems. Potato plants of the susceptible cultivar ‘Ilam Hardy’ were grown for ∼4 weeks in a controlled growth chamber, with a 16 h photoperiod at 23°C and 80% humidity (to a height of ∼20 cm). Plants were inoculated under the second fully expanded leaf by injecting 10 μL of bacteria grown in LB into the stem with a micropipette (either 10^4^ or 10^6^ cells per inoculation site). A total of 22 plants were used for each treatment. Inoculation sites were covered with plastic wrap to avoid desiccation and the plants were incubated in growth chambers at 23°C and high humidity (>80%). The length of blackleg lesions was measured on each plant daily for a period of 14 days post-inoculation (dpi), with absent lesions and lesions of <0.5 mm recorded as ‘<0.5.’

Data from the blackleg assays were primarily explored graphically. Statistical analysis was carried out for the data at day 14. For the final assessment at day 14, the percentage of plants showing measurable lesions (measurable is lesions not recorded as <0.5) was analyzed with a binomial generalized linear model with a logit link (McCullagh and Nelder, 1989). Predicted percentages and associated 95% confidence limits were obtained on the logit scale and back-transformed to percentages. Also for the final assessment, the length of lesions for plants with measurable lesions was analyzed with ANOVA. Mean lesion length was obtained, along with 95% confidence limits for the means. The strains were compared within the analysis of deviance/variance, and were assessed with a X^2^/*F*-test. All analyses were carried out with GenStat (GenStat Committee, 2012).

### Detection of Loci Belonging to the *cfa* Biosynthetic Cluster and HAI2 in Strains of *Pectobacterium*

The collection of *Pectobacterium* strains was screened for the presence of *cfa6* and *cfa7* by PCR. Six other loci associated with HAI2 (Vanga et al., 2012) were targeted by PCR. These included *attL* and *attR*, the direct repeats that delineate HAI2 when the ICE is integrated in the chromosome of *P. atrosepticum* SCRI1043 (at the 3′ end of a *phe*-tRNA gene between the CDS identifiers *ECA0515* and *ECA0615*). The presence of *ECA0516* (*soj*), *ECA0525* (*topB*), *ECA0532* (*pilL*) and *ECA0614* (*int*), four genes predicted to be involved in the stable maintenance, excision, and transfer of HAI2 (Vanga et al., 2012), was also examined. The primers used for amplification of the eight loci are listed in **Table 1**. PCR was performed using *Taq* polymerase (Roche Diagnostics) in a GeneAmp^R^ PCR System 9700 thermocycler (Applied Biosciences) with the following steps: 5 min at 95°C for initial denaturation, followed by 35 amplification cycles of 94°C for 30 s, annealing for 30 s, and 72°C for 60 s and a final extension phase at 72°C for 7 min. The annealing temperatures for each PCR are described in **Table 1**.

**Table 1:** Primers used in this study.

Target locus	Primer name	Primer sequence (5′–3′)	Annealing temperature (°C)	Size of amplicon (bp)
**Primers used to detect the *cfa* biosynthetic genes and other loci associated with HAI2**				
*attL*	LE1	GATTCGTGGGGTGATTAAGG	57	470
	LE2	CCGCCCTTTGTCGAAATTA		
*attR*	RE2	TACGATGAAGCGAGAGCACA	59	753
	RE1	ACGTAGCTCAAGCCAGTCGT		
*ECA0516*	ECA0516F	CTGAAAGGGTGAGTCGCT	57	1146
	ECA0516R	CACTGGAATGGATGCTTGG		
*ECA0525*	ECA0525F	CACCCGTTGGTATTAAAGCG	57	2232
	ECA0525R	CATCTGTTGGGCTGGTAG		
*ECA0532*	pilLF	GTGCAACGACCCCTGTATCT	59	542
	pilLR	TAGAGCGGCGTATCAACCTT		
*ECA0614*	intF	ATGTCAGATAAGTCAATGACC	58	1022
	intR	TTACATTACTGTCAAGTCAT		
*cfa6*	CFA6F	ACCAGTATTTGGCGTTGAGG	60	421
	CFA6R	GTCGCTACAAGATGCGATCA		
*cfa7*	CFA7F	ATCGAGCTGGCATTCTGAGT	60	401
	CFA7R	CAATATCGGCCATACCCAAC		
**Primers used to detect mobilization of PbN1_GI15^∗^**				
*attB^speC^*	ATTBF	CACGCCATAAACGAAAAACC	51	152
	ATTBR	TGGCCAATACGGTTTAATGC		
*attP*^PbN1_GI15^	ATTPF	TAACCGCCCGTTAAGCTTCT	52	154
	ATTPF	TCAGGATGTATATGCTCACAC		
*odc*	ODCF	GCAGCAGGCTGGCTTCTCCC	60	170
	ODCR	GACGTCTAGCGCGGCGAACA		
*cfa7*	CFA7PbrF	GGCCACTGCGACCCTTGACC	60	166
	CFA7PbrR	TTTCCCTACCGAGCCGCCGT		

*^∗^The *attL* and *attR* loci (both 153 bp) from PbN1_GI15 were amplified using an annealing temperature of 52°C using primers ATTPF and ATTBR and ATTBF and ATTPR, respectively.*

### qPCR Assays to Measure Excision of HAI2-Related ICEs in *Pectobacterium*

Quantitative PCR (qPCR) was used to measure excision of HAI2-related ICEs in different *Pectobacterium* isolates by quantifying the amounts of *attP, attB^*0515*^* and a reference gene located on the core genome, upstream of the ICE (Vanga et al., 2012). An *attP* locus is formed upon the circularization of an ICE during excision and the subsequent formation of an extrachromosomal element. Excision of the ICE results in the reconstitution of the chromosomal insertion site, *attB^*0515*^*, located within the *phe*-tRNA gene downstream of *ECA0515* in *P. atrosepticum* SCRI1043 (and other *Pectobacterium*). Thus, detection of *attB^*0515*^* occurs when the ICE is not inserted in this target site on the chromosome.

Excision assays were performed by growing each strain in triplicate for 24 h in MM. A single DNA sample was then extracted from each culture using the DNeasy tissue kit (Qiagen). A qPCR was performed in triplicate for each DNA sample using the iTaq SYBR Green Supermix with ROX (Biorad) and variable concentrations of each primer (Vanga et al., 2012). Amplification was performed by the 7500 Fast System (Applied Biosystems) using the following conditions: 2 min 30 s at 95°C for initial denaturation, followed by 40 cycles of 95°C for 15 s, annealing for 20 s, and 72°C for 20 s. The annealing temperatures used for *attB^*0515*^, attP*, and *ECA0515* are described by Vanga et al. (2012). To check the integrity of the qPCR products, melting curves were carried out for each reaction using the following conditions: 15 s at 95°C, followed by annealing at reaction-specific annealing temperatures for 60 s and extension at 72°C for 1 min.

The Ct values for each qPCR were collected and the mean Ct produced by the three replicate reactions for each DNA sample was calculated using the Applied Biosystems 7500 Fast System SDS software V. 1.3. The amplification efficiency of all assays was also determined using plasmid pBP515, containing single copies of *attP, attB^*0515*^*, and *ECA0515*, linearized with *Spe*I. Serial dilutions of the plasmid were prepared and used as templates for qPCR to generate standard curves for each of the PCRs by plotting DNA concentration versus log (Ct) value. Amplification efficiency was determined using the slope values obtained from the standard curves and the equation *E* = 10^(-1/slope)^. The ratios of *attB^*0515*^* and *attP* (R*_att_*) to *ECA0515* were then calculated for each qPCR, by normalizing the results for *attB^*0515*^* and *attP* against those obtained using *ECA0515*, with correction for differences in amplification efficiencies (Vanga et al., 2012).

The log_10_ ratios for *attB^*0515*^* and *attP* were analyzed with ANOVA (the log_10_ transformation was used to stabilize for variance). Differences between the mean log_10_ ratios for each isolate and the mean for *P. atrosepticum* SCRI1043 were calculated and back-transformed, to give REST (relative expression) ratios between each isolate (‘sample’) and *P. atrosepticum* SCRI1043 (‘control’). To identify significant differences between DNA samples from different strains of *Pectobacterium*, the back-transformed least significant difference (LSD) was then calculated to give a least significant ratio (LSR); that is the smallest ratio for which REST is significantly greater than 1 (1/LSR gives the largest ratio for which REST is significantly smaller than 1). All analyses were carried out with GenStat Committee (2012).

### Genome Sequencing of HAI2-Related ICEs Encoding Cfa in *Pectobacterium*

To characterize ICEs harboring the *cfa* biosynthetic cluster, the genomes of *P. atrosepticum* ICMP 1526, the type strain for *P. atrosepticum*, and *P. carotovorum* subsp. *brasiliensis* ICMP 19477 were sequenced using a Roche FLX 454 sequencer by the Liverpool Advanced Genomics Facility (Liverpool University, UK). Sequences were assembled with Newbler to produce draft genome sequences to 40–50 × coverage, comprising 38 and 35 contigs for *P. atrosepticum* ICMP 1526 and *P. carotovorum* subsp. *brasiliensis* ICMP 19477, respectively (Panda et al., 2015). The genome of UGC32 was also sequenced using the HiSeq 2000 system from Illumina sequencing services to generate pair-end reads with a genome coverage of ∼60× (Panda et al., 2015). UGC32 was previously identified as a *P. carotovorum* subsp. *carotovorum* isolate with the capacity to cause blackleg (Slawiak and Lojkowska, 2009). The contigs for each strain were ordered and orientated with respect to the reference genome sequence of *P. atrosepticum* SCRI1043 (accession BX950851) using MUMmer. Annotation of the genome sequences was completed using the PGAAP pipeline^1^. The genome sequencing, assembly and annotation of SCRI1043ΔHAI2 were performed as described for UGC32 and gave the following statistics: number of contigs/scaffolds, 135; N50, 1,156,993.

### Comparative Analysis of HAI2-Related ICEs Encoding Cfa in *Pectobacterium*

Geneious Pro 5.5.5 (Kearse et al., 2012) was used to collate genome sequence data for each strain, and the Blast suite version 2.2.31 (blastn using max E value 1e-1) was used to identify *cfa* biosynthetic clusters in each genome (using the *cfa* genes from *P. atrosepticum* SCRI1043 as a reference). The genetic structure and organization of the *cfa* clusters and their related ICEs were then curated manually from the annotated genomes of each strain by conducting blastx comparisons of individual CDSs with HAI2 from *P. atrosepticum* SCRI1043. Each ICE was delineated by the presence of direct repeats *attL* and *attR*. DNA sequences for the ICEs can be obtained from the annotated draft genome sequences of *P. atrosepticum* ICMP 1526 and *P*. *carotovorum* subsp. *brasiliensis* ICMP 19477, which have the Genbank accessions ALIV00000000 and ALIU00000000, respectively. The accession number for the draft genome of *P. carotovorum* subsp. *carotovorum* UGC32 is AODU00000000. Similarities and differences in the HAI2-related ICEs were visualized in Easyfig 2.2.2 (Sullivan et al., 2011) by performing tblastx comparisons with a cut off E value of 1e-10.

### Excision of PbN1_GI15 in *P*. *carotovorum* subsp. *brasiliensis* ICMP 19477

Integration of PbN1_GI15 into the chromosomal target site *attB^speC^* in *P. carotovorum* subsp. *brasiliensis* ICMP 19477 was examined by amplifying the *attL* (153 bp) and *attR* (153 bp) sites using primers ATTPF and ATTBR and ATTBF and ATTPR, respectively. Excision of PbN1_GI15 was also studied by PCR to establish whether PbN1_GI15 could mobilize from the chromosome of this bacterium. Initially, a PCR was performed using primers ATTBF and ATTBR to detect the 152-bp amplicon indicative of reconstitution of the chromosomal target site *attB^speC^* upon mobilization of the putative ICE. A PCR strategy was then designed to amplify a 154-bp PCR product (*attP*^PbN1_GI15^) using primers ATTPF and ATTPR only when the *attL* and *attR* sites delineating PbN1_GI15 recombined during excision to form an *attP* site indicative of circularization. To confirm DNA extracted from *P*. *carotovorum* subsp. *brasiliensis* ICMP 19477 could be amplified, primers were designed to amplify the ornithine decarboxylase gene (*odc*) located on the core genome of this bacterium. The *cfa7* gene was also targeted by PCR to re-confirm the presence of the *cfa* gene cluster. All primers are described in **Table 1**. PCR amplification conditions consisted of an initial denaturation step at 95°C for 3 min, followed by 40 cycles of 94°C for 30 s, an annealing temperature specific to each primer pair (**Table 1**) for 30 s, and an extension step at 72°C for 30 s. A final extension step was carried out at 72°C for 5 min. Negative PCR controls were included for PCRs with each primer set.

## Results

### HAI2 Is Required for Full Virulence of *P. atrosepticum* on Potato Stems

A role for HAI2 in virulence of *P. atrosepticum* SCRI1043 was examined by comparing the aggressiveness of the wild type with that of a strain in which the ICE had been removed by CRISPR-Cas-mediated genome targeting (Vercoe et al., 2013; Richter et al., 2014). The HAI2 junction of this deletion mutant had been previously sequenced. To confirm this deletion and to rule out off-target mutations of this ‘ICE-less’ strain (SCRI1043ΔHAI2), we sequenced the genome of the mutant. Assembly of the sequenced genome showed that the only large scale deletion from the genome was that of the ∼100 kb ICE HAI2 targeted using the CRISPR-Cas system (Supplementary Figure S1), demonstrating the specificity of the endogenous type I-F CRISPR-Cas system for genome editing.

**FIGURE 1:**
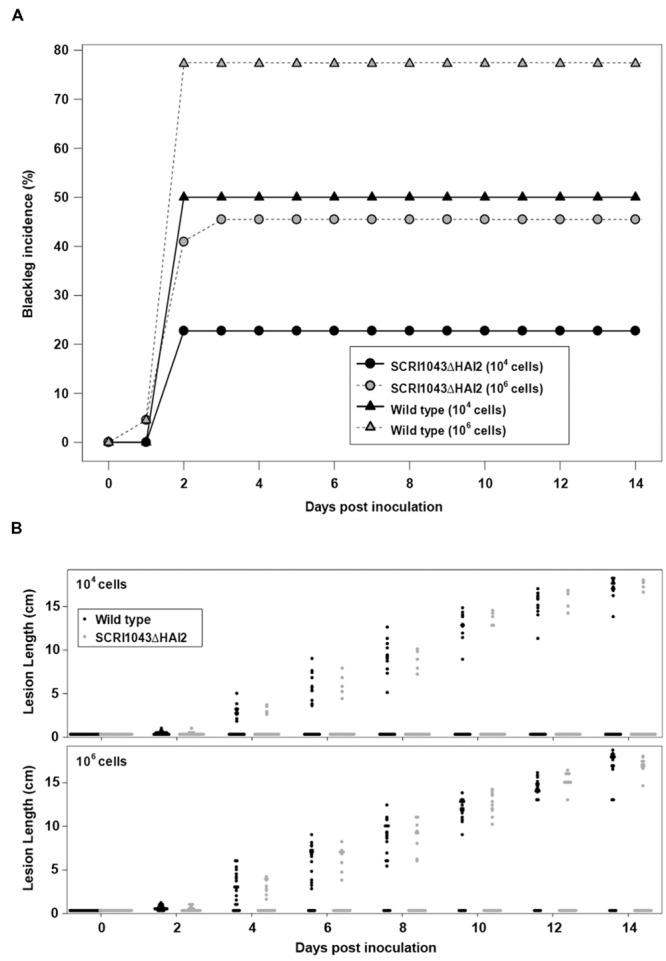
**Deletion of HAI2 from *Pectobacterium atrosepticum* SCRI1043 reduces development of blackleg on potato stems. (A)** Incidence of blackleg (% of plants with stem lesions) at each assessment for plants inoculated with either SCRI1043ΔHAI2 or the wild type. Each bacterium was inoculated into plants at either 10^4^ or 10^6^ cells per inoculation site. **(B)** Dot-histograms of lesion lengths on stems for each of 22 potato plants inoculated with either 10^4^ or 10^6^ cells per inoculation site of SCRI1043ΔHAI2 or the wild type. Measurements were taken every second day to 14 days post-inoculation.

Plant infection assays were subsequently performed with the ‘ICE-less’ strain or the wild type using two different starting quantities of inoculum. In the wild type, blackleg incidence increased with greater inoculum (*p* = 0.018): 77.3% (95% confidence limits; 55.7, 90.2) of plants showing symptoms when inoculated with 10^6^ cells versus 50.0% (30.2, 69.8) when inoculated with 10^4^ cells per inoculation site (**Figure 1A**). The concentration of the inoculum also had a significant effect on the incidence of blackleg in plants inoculated with the ‘ICE-less’ strain, with blackleg incidence reaching 45.5% (95% confidence limits; 26.5, 65.9) in plants inoculated with 10^6^ cells of the ICE-less strain but only 22.7% (9.8, 44.3) when inoculated with 10^4^ cells after 14 days (**Figure 1A**).

Using either inoculum amounts, significantly more plants developed blackleg lesions when inoculated with the wild type compared with the ‘ICE-less’ strain (*p* = 0.005; **Figure 1A**). For plants that formed lesions, however, the lesions were similar in length whether inoculated with the wild type or the ‘ICE-less’ strain. The amount of inoculum did not alter the length of these lesions either (*p* > 0.4 for the strain and concentration main effects and for the interaction between them at 14 dpi; **Figure 1B**). Thus, the HAI2 mutant produced a lower incidence of blackleg, but once disease symptoms became established the mutation had no detectable impact on lesion development.

### Detection of HAI2 and HAI2-Related ICEs in Isolates of *P*. *atrosepticum*

Given the role of HAI2 in the virulence of *P. atrosepticum* SCRI1043, the distribution of HAI2-like loci was examined in other potato isolates of *P. atrosepticum* by PCR. Products indicative of *cfa6* and *cfa7* were amplified using DNA from all nine isolates (Supplementary Table S1). Amplicons were also generated using primers targeted to *ECA0516, ECA0525, ECA0532*, and *ECA0614* (indicative of the presence of HAI2) using DNA from all isolates. These data suggested that not only was the *cfa* cluster ubiquitous in this pathogen, but it was likely to be harbored on similar ICEs in different strains.

To examine whether the ICEs carrying the *cfa* cluster had integrated into the *phe*-tRNA gene immediately downstream of *ECA0515* (*attB^*0515*^*) in each strain (as in SCRI1043), attempts were made to amplify *attL* and *attR* by PCR. The *attL* and *attR* loci were detected in all *P. atrosepticum* strains with the exception of ICMP 1526 (the type strain for *P. atrosepticum*; Supplementary Table S1). The inability to detect these loci in *P. atrosepticum* ICMP 1526 suggested that the regions close to the proximity of the ICE were sufficiently different from those in HAI2 to restrict binding of the PCR primers used for PCR amplification. Alternatively, the *cfa* cluster was located on a plasmid or HAI2 had integrated at a chromosomal target site other than *attB^*0515*^* in *P. atrosepticum* ICMP 1526.

**FIGURE 2:**
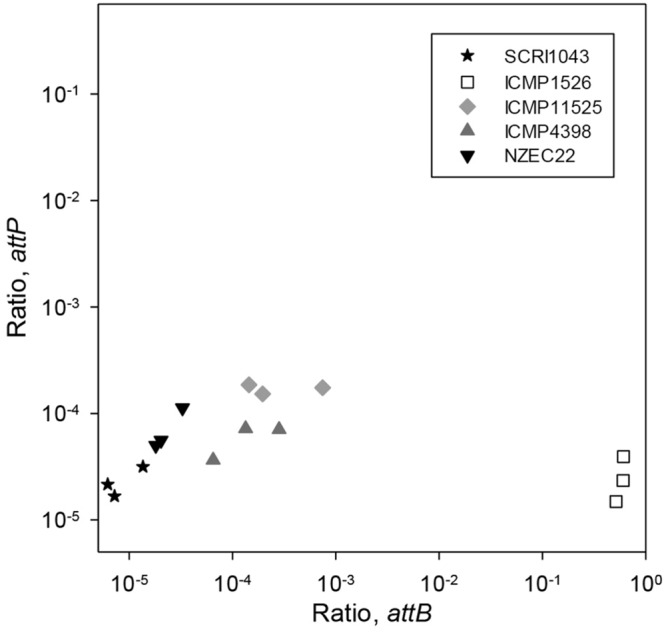
**Excision of HAI2-like elements in various strains of *P. atrosepticum* grown *in vitro*.** Back-transformed mean log^10^ ratios (normalized to *ECA0515*) for *attP* are plotted against those for *attB^*0515*^* on a log10 spaced axes for *P. atrosepticum* strains SCRI1043, NZEC22, ICMP 11525, ICMP 4398, and ICMP 1526. Cultures were grown in MM for 24 h, in triplicate. Three qPCR reactions were performed for each DNA sample and the mean ratio was plotted. The LSD between two means on the transformed scale was 0.29 for *attP* and was 0.44 for *attB* (at the 5% significance level with 10 degrees of freedom).

At the same time, the formation of *attP* and *attB^*0515*^* was measured by qPCR to determine the dynamics of ICE excision in *P. atrosepticum*. In these experiments, both *attP* and *attB^*0515*^* were detected in all isolates (**Figure 2**). The ratios for *attP* and *attB^*0515*^* were 2.25 × 10^-5^ and 8.45 × 10^-6^ in *P. atrosepticum* SCRI1043, consistent with the low frequency of HAI2 excision from the *phe*-tRNA gene immediately downstream of *ECA0515* and the formation of the circular extrachromosomal form reported previously (Vanga et al., 2012, 2015). The relative proportion of *attP* compared to *attB^*0515*^* calculated using these ratios was 2.67. A ratio above 1 suggested that the ICE may be replicating at low copy number.

Consistent with the findings in *P. atrosepticum* SCRI1043, in *P. atrosepticum* NZEC22 the relative proportion of *attP* (2.29 × 10^-5^) to *attB^*0515*^* (6.81 × 10^-5^) was 2.97. In *P. atrosepticum* ICMP 4398 and ICMP 11525, however, the relative proportions of *attP* to *attB^*0515*^* were 0.42 and 0.62, respectively. A value lower than 1 suggested that in these two strains the ICE was either highly unstable or was capable of integrating into a second chromosomal target site as well as *attB^*0515*^*. Furthermore, based on the *attP* and *attB^*0515*^* ratios, ICE excision in the two strains was ∼10-fold greater than for *P. atrosepticum* SCRI1043 (*p* ranging from *p* < 0.001 to *p* < 0.05).

In *P. atrosepticum* ICMP 1526, *attP* (2.39 × 10^-5^) was similar (*p* > 0.05) to that for *P. atrosepticum* SCRI1043, but *attB^*0515*^* was detected in almost all cells (CT*_attB_* = Ct*_*ECA0515*_*; **Figure 2**). Given that *attL* and *attR* were not detected in *P. atrosepticum* ICMP 1526 by PCR (Supplementary Table S1), and that *attB^*0515*^* was detected in most cells of *P. atrosepticum* ICMP 1526, HAI2 was almost certainly integrated elsewhere in the genome of this strain.

### Detection of HAI2-Related ICEs in Isolates of *P*. *carotovorum* Causing Blackleg

The distribution of HAI2-like ICEs and the *cfa* biosynthetic genes was also examined in 62 *P. carotovorum* isolates from potato by PCR. A *cfa6* amplicon and a *cfa7* product were amplified using DNA from only seven (Supplementary Table S1). These isolates were all known to cause blackleg (Slawiak and Lojkowska, 2009; Panda et al., 2012), and included the New Zealand isolates *P. carotovorum* subsp. *brasiliensis* ICMP 19477 and NZEC150, as well as the five *P. carotovorum* subsp. *carotovorum* strains from Peru that were already known to harbor the *cfa* biosynthetic cluster (Slawiak and Lojkowska, 2009). The *attL, ECA0516, ECA0525*, and *ECA0532* fragments were not amplified using DNA from any of the *P. carotovorum* isolates, while *attR* and *ECA0614* were only amplified from *P. carotovorum* subsp. *carotovorum* UGC32 and the other related isolates from Peru. Consistent with these results, *attP* was not detected using DNA from *P. carotovorum* in qPCR (data not shown).

### The ICE Encoding Cfa in *P. carotovorum* subsp. *brasiliensis* ICMP 19477 Is Distinct from HAI2

The absence of the HAI2-related loci in isolates such as *P. carotovorum* subsp. *brasiliensis* ICMP 19477 suggested that the *cfa* cluster in *P. carotovorum* was located on a mobile element distinct from HAI2. Thus, the genetic structure and organization of the *cfa* biosynthetic clusters in *P. atrosepticum* and *P. carotovorum* as well as the mobile elements encoding them were examined further by sequencing the genomes of *P. atrosepticum* ICMP 1526, *P. carotovorum* subsp. *brasiliensis* ICMP 19477 and *P. carotovorum* subsp. *carotovorum* UGC32. *P. atrosepticum* ICMP 1526 was chosen for DNA sequencing, as it was the type strain for *P. atrosepticum* and *attL* and *attR* were not amplified using DNA from this isolate as a template in PCR. These data, along with the high amount of *attB^*0515*^* detected in qPCR, suggested an alternative HAI2 insertion site in *P. atrosepticum* ICMP 1526. PCR also failed to amplify *attL, attR*, and *attP* from *P. carotovorum* subsp. *brasiliensis* ICMP 19477, which causes blackleg and represents one of the two lineages of *P. carotovorum* on potato. Similarly, the DNA sequence of UGC32 was obtained as this isolate has the capacity to cause blackleg and is purportedly characteristic of the second lineage of *P. carotovorum* (*P. carotovorum* subsp. *carotovorum*).

**FIGURE 3:**
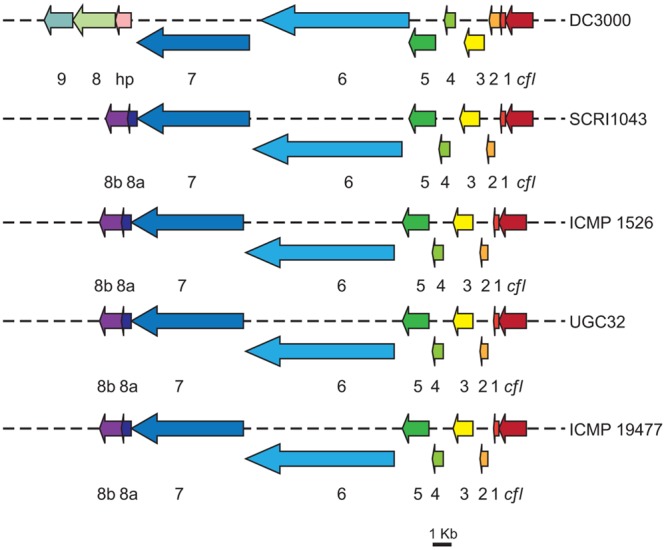
**Organization of the *cfa* biosynthetic operons in different isolates of *Pectobacterium*.** The *cfa* (1–9) and the *cfl* genes are illustrated (where appropriate) for *Pseudomonas syringae* pv. tomato DC3000, *P. atrosepticum* SCRI1043, *P. atrosepticum* ICMP 1526, *P. carotovorum* subsp. *carotovorum* UGC32 and *P. carotovorum* subsp. *brasiliensis* ICMP 19477. Colored arrows represent the predicted biosynthetic genes [scale bar: 1 Kb], hp, denotes a gene encoding a hypothetical protein predicted to have thioesterase activity, which has weak similarity to *cfa8a*. The *cfa9* gene is found only in *P. syringae* pv. tomato DC3000.

Comparison of HAI2 from *P. atrosepticum* SCRI1043 and the draft genome sequences from *P. atrosepticum* ICMP 1526, *P. carotovorum* subsp. *brasiliensis* ICMP 19477, and *P. carotovorum* subsp. *carotovorum* UGC32 revealed homologous *cfa* biosynthetic clusters in each of the *Pectobacterium* isolates (**Figure 3**). Their organization was highly conserved, each cluster spanning 22 kb and comprising the nine genes required for synthesis of the polyketide in *P. atrosepticum* SCRI1043 (*cfa1* to *cfa8b*). The amino acid sequences of the *cfa* genes also had between 100 and 96% identity to those in *P. atrosepticum* SCRI1043 (100% for *P. atrosepticum* ICMP 1526 and 96% for both *P. carotovorum* subsp. *brasiliensis* ICMP 19477 and *P. carotovorum* subsp. *carotovorum* UGC32). A *cfl* gene, which encodes coronafacate ligase, was located adjacent to the *cfa* biosynthetic clusters in each strain, although the amino acid sequences they encode were highly divergent; the amino acid identity of *cfl* in *P. carotovorum* subsp. *carotovorum* UGC32 (when compared with the *cfl* gene in *P. atrosepticum* SCRI1043) was 93%, but only 35% in *P. atrosepticum* ICMP 1526 and 31% in *P. carotovorum* subsp. *brasiliensis* ICMP 19477. As in *P. atrosepticum* SCRI1043, however, the *cma* biosynthetic cluster was absent from the genomes of the three newly sequenced pathogens. As coronafacate ligase mediates conjugation of Cfa to Cma in pseudomonads, the absence of a *cma* biosynthetic cluster and the divergence of the *cfl* gene suggests that Cfa may be ligated to one or more unidentified molecules in *Pectobacterium*.

The homologous *cfa* biosynthetic clusters in each of the *Pectobacterium* isolates were also highly similar to the *cfa* cluster from *P. syringae* pv. tomato DC3000 (**Figure 3**). Amino acid sequence comparisons showing 45–75% identity between proteins Cfl to Cfa8b from *P. atrosepticum* SCRI1043 and those (Cfl to Cfa8) in the pseudomonad. In contrast, *cfa9* was not detected in the genomes of the *Pectobacterium* isolates. Cfa9 is dispensable for Cfa and Cor production in *P. syringae* pv. tomato DC3000, but may increase the release of enzyme-bound products from the Cor pathway (Rangaswamy et al., 1998).

**FIGURE 4:**
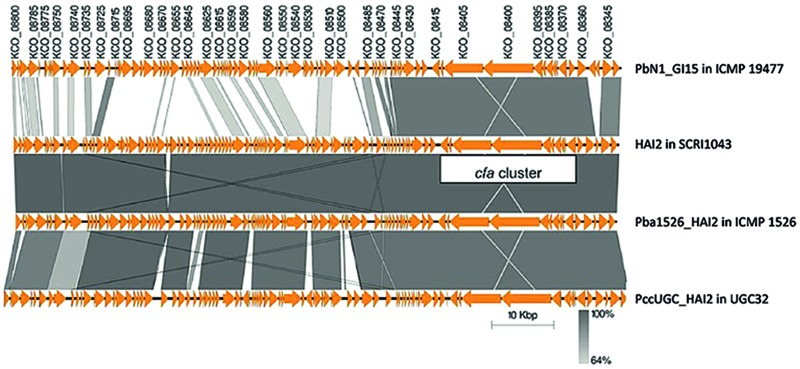
**A nucleotide alignment of putative ICEs encoding coronafacic acid in strains of *P. atrosepticum* and *P. carotovorum*.** The schematic representation drawn using Easyfig (Sullivan et al., 2011) shows pairwise alignments between PbN1_GI15 from *P. carotovorum* subsp. *brasiliensis* ICMP 19477, HAI2 from *P. atrosepticum*, SCRI1043, Pba1526_HAI2 from *P. atrosepticum* ICMP 1526 and PccUGC_HAI2 from *P. carotovorum* subsp. *carotovorum* UGC32. Orange arrows indicate annotated Coding Domain Sequences (CDSs). The locus tags for CDSs from the putative ICE in *P. carotovorum* subsp. *brasiliensis* ICMP 19477 are provided above the arrows, and their regions of nucleotide identity with CDSs from other putative ICEs are represented by gray shading. The genome coordinates for the ICEs in each strain are as follows: 910293 to 1008796 for PbN1_GI15, 590755 to 688630 for HAI2, 958243 to 1055429 for Pba1526_HAI2 and 591326 to 692291 for PccUGC_HAI2.

As in *P. atrosepticum* SCRI1043, the *cfa* biosynthetic clusters in *P. atrosepticum* ICMP 1526, *P. carotovorum* subsp. *brasiliensis* ICMP 19477 and *P. carotovorum* subsp. *carotovorum* UGC32 were located on putative ICEs. As predicted from excision assays, the ICE (named Pba1526_HAI2) in *P. atrosepticum* ICMP 1526 had high sequence identity (99%) to HAI2 (**Figure 4**), but was inserted into a second *phe*-tRNA gene located adjacent to *speC* (locus tag: *ECA0967*). Pba1526_HAI2 was 97,216 bp, contained 97 putative CDSs (Supplementary Table S2) and was delineated by 49-bp direct repeat sequences *attL* and *attR*. Gene organization on Pba1526_HAI2 was almost identical to that on HAI2 (**Figure 4**; Supplementary Table S2). Synteny was lost in only one region, notably, within and immediately adjacent to *pilV*, which encodes a component of the type IV pilus involved in conjugation. The *pilV* shuﬄon on other mobile elements acts as a biological switch, selecting one of seven C-terminal segments of the *pilV* gene that confer recipient specificity during conjugation (Komano et al., 1987).

The *cfa* cluster was harbored on two different ICEs in *P. carotovorum*. PccUGC_HAI2, the ICE in *P. carotovorum* subsp. *carotovorum* UGC32, was highly similar to HAI2 (95% nucleotide identity) and located within the same target site (*attB^*0515*^*). PbN1_GI15, the ICE in *P. carotovorum* subsp. *brasiliensis* ICMP 19477, was distinct from HAI2 and was inserted within the *phe*-tRNA gene located adjacent to *speC*. Both PccUGC_HAI2 and PbN1_GI15 were delineated by the 49-bp direct repeats that define HAI2 and Pba1526_HAI2, which are important for insertion into either of the two target sites.

PccUGC_HAI2 was 100,966 bp in size and contained 100 putative CDSs. Of these 100 CDSs, 90 were present in the related ICE, HAI2. The functions of the 10 CDSs unique to PccUGC_HAI2 were unknown as they encode hypothetical proteins. The exception was GO33_02925, which possesses a domain with similarity to Abi_2 family proteins that include the bacteriophage resistance AbiD1 protein from *Lactococcus lactis* (Anba et al., 1995). PccUGC_HAI2, on the other hand, was missing eight CDSs from HAI2 including two genes (*ECA0582* and *ECA0583*) that appear to encode a putative toxin–antitoxin system with similarity to the *pemK*-*pemI* addiction module. The protein encoded by *ECA0582* had 28% amino acid identity to the factor encoded by *pemK*, which stabilizes plasmid R100 in bacterial populations by killing plasmid-free segregants (Tsuchimoto et al., 1992). *ECA0583* produced a protein with 33% similarity to PemI, an unstable inhibitor of PemK that suppresses plasmid stabilization. No alternative toxin–antitoxin system could be identified on PccUGC_HAI2 using RASTA-Bacteria: a web-based tool for identifying toxin–antitoxin loci in prokaryotes (Sevin and Barloy-Hubler, 2007).

PbN1_GI15 was 98,504 bp and possessed a total of 93 putative CDSs (Supplementary Tables S2 and S3). Although it was distinct from HAI2, many of the CDSs in PbN1_GI15 had strong similarity to those in HAI2, especially in the region surrounding the *cfa* cluster between KCO_08485 (*ECA0587* in HAI2) and KCO_08340 (*ECA0614* in HAI2; **Figure 4**; Supplementary Table S2). Of the 28 putative CDSs annotated in this region of PbN1_GI15, 24 were similar to genes from HAI2, including the integrase (locus tag KCO_08340) that was located adjacent to one of the direct repeats (*attR*) delineating the ICE. The integrase mediates excision of HAI2 out of the chromosome (Vanga et al., 2015). *ECA0612*, which encoded a DinB-family protein involved in regulation of SOS-related responses, was absent from PbN1_GI15, with two putative CDSs (KCO_08350 and KCO_08355) transcribed on the complementary strand of PbN1_GI15 instead. Like ECA0612, KCO_08350 possessed a DNA-binding motif and is predicted to be involved in transcriptional regulation.

Sequence similarities between PbN1_GI15 and HAI2 were dramatically lower upstream of KCO_08485, which encodes a second putative integrase. Of most note, a cluster of seven genes was present in HAI2 but absent from PbN1_GI15. This cluster encoded the same *pemIK* putative toxin–antitoxin (*ECA0582-83*) system missing from PbN1_GI15 as well as a Type I restriction-modification system encoded by *ECA0584* (methylase) and *ECA0585* (restriction endonuclease). As for PccUGC_HAI2, no alternative toxin–antitoxin (or restriction modification) system could be identified on the ICE using RASTA-Bacteria (Sevin and Barloy-Hubler, 2007).

A second cluster of genes was absent in PbN1_GI15. These genes were located adjacent to the *pil* operon in HAI2 and were predicted to encode proteins involved in conjugal transfer (e.g., TraE). In contrast, PbN1_GI15 harbored a group of six CDSs (locus tags KCO_08550 to KCO_08525) that were absent from HAI2 that have largely no known function. The only exception was KCO_08530, which was predicted to produce an SMF protein likely involved in DNA uptake. PbN1_GI15 also encoded a RNA-dependent DNA polymerase similar to those associated with group II introns (encoded by KCO_08725) as well as a distinct type IV secretion system.

### Mobilization of PbN1_GI15 in *P*. *carotovorum* subsp. *brasiliensis* ICMP 19477

**FIGURE 5:**
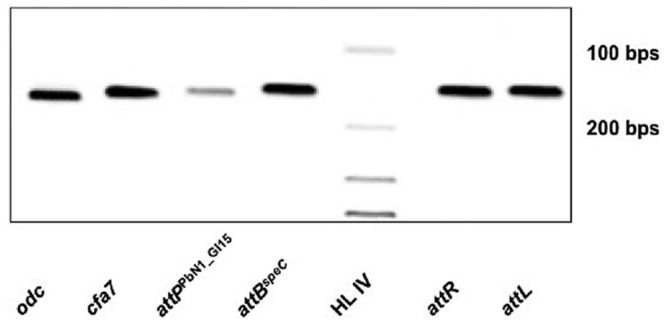
**Excision of ICE PbN1_GI15 in *P. carotovorum* subsp. *brasiliensis* ICMP 19477.** Electrophoresis gel image showing PCR amplicons associated with mobilization of ICE PbN1_GI15. Lane 1 (*odc*), ornithine decarboxylase gene; lane 2 (*cfa7*), coronafacic acid biosynthetic gene; lane 3 (*attP*^PbN1_GI15^), the *attP* site formed upon the circularization of PbN1_GI15 during excision from *attB^speC^*; lane 4 (*attB^speC^*), 152-bp amplicon indicative of reconstitution of the chromosomal target site *attB^speC^* upon mobilization of PbN1_GI15; lane 5 (HL IV), Hyperladder IV (Bioline, UK); lane 6 (*attR*), the 153 bp sequence delimiting the right end of PbN1_GI15 when integrated within the *attB* site adjacent the *speC*; lane 7 (*attL*), the 153 bp sequence delimiting the left end of PbN1_GI15 when integrated within the *attB* site adjacent to *speC*.

Despite the differences between PbN1_GI15 and HAI2, PbN1_GI15 retains features of a mobile element (e.g., an integrase, direct repeats). Thus, mobilization of PbN1_GI15 was examined by PCR. Firstly, integration of PbN1_GI15 into the chromosomal target site *attB^speC^* in *P. carotovorum* subsp. *brasiliensis* ICMP 19477 was confirmed, with amplicons indicative of both *attL* and *attR* produced (**Figure 5**). However, many ICEs become anchored in the genome and lose their capacity to excise. Therefore, the excision of PbN1_GI15 from the chromosome was monitored by measuring the formation of *attP*^PbN1_GI15^ and *attB^speC^* using PCR. These reactions produced fragments indicative of both loci (**Figure 5**), confirming precise excision of PbN1_GI15 and reconstitution of the chromosomal target site in *P. carotovorum* subsp. *brasiliensis* ICMP 19477. The amplification of *attP*^PbN1_GI15^ was consistent with this ICE retaining the capacity to excise from the chromosome prior to transfer to donor cells. No amplicons were produced in the negative PCR controls (containing no template DNA).

## Discussion

Deletion of HAI2 from the genome of *P. atrosepticum* SCRI1043 by CRISPR-Cas-mediated genome editing resulted in reduced virulence of the pathogen on potato. Previous studies of ICE biology have relied largely on the inactivation of the virulence determinants harbored on the mobile element or on screening a population for individuals in which the ICE has been lost. Targeted inactivation of virulence determinants requires complex cloning strategies and only reveals the roles of specific genes, which can overlook many unknown virulence genes. For example, two prophages in the genome of *P. atrosepticum* SCRI1043 harbor no known virulence determinants, yet their deletion results in a reduction in the virulence of the pathogen (Evans et al., 2010). The low frequency of both ICE excision (between 10^-5^ and 10^-6^ per cell; Buchrieser et al., 1998; Lesic et al., 2004; Sakellaris et al., 2004) and subsequent loss from the bacterial population under laboratory conditions (Lovell et al., 2009) also makes the identification of strains without an ICE challenging.

The reduction in the virulence of *P. atrosepticum* SCRI1043 upon deletion of HAI2 was not entirely unexpected, given that a transposon insertion within the *cfa* biosynthetic cluster reduces severity of blackleg in plants infected by the pathogen (Bell et al., 2004). Inactivation of PbTopo IIIβ, a topoisomerase encoded on HAI2, also reduces the incidence of the disease (Vanga et al., 2012). However, the deletion of the entire ICE did not result in the reduction in the length of blackleg symptoms described by Bell et al. (2004), which may have been because a different cultivar was used in the two plant assays or as a result of different experimental conditions. Alternatively, it may be due to hidden costs associated with retaining the ICE. ICEs can have great evolutionary benefits, but may also be disadvantageous to the cell. For example, in susceptible leaf tissues of *Phaseolus vulgaris, P. syringae* pv. *phaseolicola* exhibits reduced colony formation compared with an isogenic strain without the ICE PPHGI-1 (Godfrey et al., 2010). Reduced colony formation of the strain containing the PPHGI-1 is predicted to result from a compromise in the *in planta* fitness of the bacterium due to one or more plant responses triggered by the ICE.

Consistent with the findings of Slawiak and Lojkowska (2009), a screen of isolates of *P. atrosepticum* using PCR revealed loci associated with the *cfa* biosynthetic cluster in all. This, together with the ubiquitous nature of other loci associated with HAI2, suggested that the ICE and the virulence factor it harbors were acquired prior to the speciation of *P. atrosepticum*. Therefore, it appears that the ICE remains under strong selective pressure to be retained. Comparative genomics of *P. atrosepticum* SCRI1043 and *P. atrosepticum* ICMP 1526 confirmed the very high level of homology and synteny amongst the ICEs in *P. atrosepticum*. Of particular note, a candidate toxin–antitoxin system was identified in both ICEs, closely associated with a putative restriction-modification system. Toxin–antitoxin systems participate in the maintenance of ICEs. For example, retention of SXT, an ICE in *Vibrio cholerae* that encodes multiple antibiotic resistance genes, is promoted by *mosA* and *mosT* (Wozniak and Waldor, 2009). When integrated in the chromosome, *mosAT* expression is shut down by MosA, but expression of *mosA* and *mosT* is up-regulated when SXT is extrachromosomal and vulnerable to loss. Thus, SXT seems to activate a toxin–antitoxin module that minimizes formation of SXT-free cells (Wozniak et al., 2009; Wozniak and Waldor, 2010). Genome context analysis has also shown that restriction-modification genes are often located on mobile elements such as plasmids and prophages (Betlach et al., 1976; Kita et al., 2003), and some are linked to recombination-related genes such as integrases, invertases, and transposases (Anton et al., 1997). Like the toxin–antitoxin system, restriction-modification systems mediate plasmid maintenance (Kulakauskas et al., 1995) and have also been implicated in site-specific recombination (Chang and Stanley, 1977). Given that HAI2 is not essential for pathogenicity of its bacterial host, it is possible that strong selection for retention of this ICE is conferred by these addiction modules.

Comparative analyses of the ICEs in *P. atrosepticum* ICMP 1526 and *P. atrosepticum* SCRI1043 revealed they can insert into the identical 49-bp *attB* sites present in either of two *phe*-tRNA. These two *phe*-tRNAs are located in different regions of the chromosome, one adjacent to *speC* (locus tag: *ECA0967*) and one some distance away (adjacent to locus tag: *ECA0615*). Insertion of HAI2 into multiple integration sites is consistent with the findings of Lovell et al. (2009), who showed that PPHGI-1 can insert into one of two sites in the genome of *P. syringae* pv. *phaseolicola* 1448A upon transmission from the donor *P. syringae* pv. *phaseolicola* 1302A. The *P. syringae* pv. *phaseolicola* 1448A genome contains two 52-bp *att* sites with the same sequence as the *att* borders of PPHGI-1 in the 1302A genome. PPHGI-1 integrated preferentially into one of these *att* sites. Interestingly, HAI2 (and its relatives) was also largely detected in the insertion site adjacent to locus tag *ECA0615*, suggesting that this ICE preferentially integrates into one insertion site in the chromosome of *P. atrosepticum*. The reasons for such a preference are unknown as the two *attB* sites in this pathogen are identical. The impacts to the cell of insertion into different sites also remain to be studied. Nevertheless, genome context may be critical for successful chromosomal insertion given that several genes on HAI2 predicted to alter DNA topology not only influence excision but also transcription of genes both on the ICE and elsewhere in the chromosome (Vanga et al., 2012, 2015). Studying the genome-wide transcription of strains in which HAI2 has been removed or inserted at different locations would identify if the ICE has different impacts on the host bacterium depending on its position in the genome.

The *cfa* biosynthetic cluster was also detected in strains belonging to two subspecies of *P. carotovorum* (as defined using multi-locus sequence analysis by Slawiak and Lojkowska, 2009 and Panda et al., 2012). *Pectobacterium carotovorum* subsp. *brasiliensis* is a well-described blackleg pathogen (Duarte et al., 2004), so, as in *P. atrosepticum*, the presence of the *cfa* cluster in ICMP 19477 may support the invasion of the xylem by this enterobacterium. A *cfa* biosynthetic cluster is not harbored in the genome of *P. carotovorum* subsp. *brasiliensis* 1692 however, (Glasner et al., 2008), indicating that Cfa production is unlikely to be essential for pathogenicity of this subspecies in stems (again, similar to its role in *P. atrosepticum*).

The capacity of *P. carotovorum* subsp. *carotovorum* to elicit blackleg disease is controversial. Thus, the detection of the *cfa* biosynthetic cluster in *P. carotovorum* subsp. *carotovorum* UGC32 and its absence from so many other isolates is intriguing. *Pectobacterium carotovorum* subsp. *carotovorum* UGC32 causes blackleg (Slawiak and Lojkowska, 2009), whereas most other *P. carotovorum* subsp. *carotovorum* isolates are only able to invade potato tubers. Perhaps, as in *P. atrosepticum* and *P. carotovorum* subsp. *brasiliensis*, this cluster supports invasion of the xylem by blackleg causing strains of *P. carotovorum* subsp. *carotovorum*. Consistent with this hypothesis, *P. carotovorum* subsp. *carotovorum* ICMP 5702 is unable to invade the stems of potato and its genome does not encode Cfa (data not shown). A broader genome-based taxonomic revision of *P. carotovorum* will be required, however, to shed further light on the importance of Cfa production to this pathogen and its capacity to cause blackleg.

The detection of *cfa* biosynthetic clusters in only limited strains of *P. carotovorum* subsp. *brasiliensis* and *P. carotovorum* subsp. *carotovorum* indicates that the clusters were probably acquired as a result of independent lateral transfer events in these strains. Consistent with this hypothesis, DNA sequencing of the ICEs harboring the *cfa* biosynthetic clusters in ICMP 19477 and UGC32 demonstrated that they were distinct from HAI2. They were also distinct from one another. The low incidence of these ICEs in *P. carotovorum* might also result from barriers to acquisition, however. For example, CRISPR-Cas systems can act as adaptive immune systems, which provide resistance to incoming mobile elements such as plasmids and phages (Dy et al., 2014). In *P. atrosepticum*, HAI2 appears to be a target for its endogenous CRISPR-Cas system except that a single nucleotide mutation adjacent to the targeted sequence in HAI2 results in CRISPR-Cas avoidance (Vercoe et al., 2013). Furthermore, genome sequencing of *P. carotovorum* subsp. *brasiliensis* ICMP 19477 and *P. carotovorum* subsp. *carotovorum* UGC32 failed to identify the candidate restriction modification and toxin–antitoxin genes in PbN1_GI15 and PccUGC_HAI2, which are present in derivatives of HAI2 in *P. atrosepticum*. As no alternative restriction-modification and toxin–antitoxin systems could be found on these ICEs either, the ICEs detected in *P. carotovorum* may be less stable than their counterparts in *P. atrosepticum*.

Finally, excision assays confirmed mobilization of PbN1_GI15 from the chromosome of *P. carotovorum* subsp. *brasiliensis* ICMP 19477. As mobilization from the chromosome occurs in preparation for transfer of an ICE to a new host (Clark and Adelberg, 1962), future acquisition of this ICE by other strains of *P. carotovorum* is possible. Furthermore, given the ICE harbors the *cfa* biosynthetic cluster, acquisition of PbN1_GI15 would be likely to enhance the capacity of recipient strains to cause blackleg. The probability of these events occurring could be addressed using transfer assays such as those described by Lovell et al. (2009).

## Author Contributions

AP, PF, KA, and CR provided substantial contributions to the conception or design of the work. AP, PF, PP, BV, RB, AL, and MF provided substantial contributions to acquisition, analysis, or interpretation of data for the work. AP, PF, KA, CR, PP, BV, RB, AL, and MF were involved in drafting the work or revising it critically for important intellectual content and provided final approval of the version to be submitted. All authors agreed to be accountable for all aspects of the work in ensuring that questions related to the accuracy or integrity of any part of the work are appropriately investigated and resolved.

## Conflict of Interest Statement

The authors declare that the research was conducted in the absence of any commercial or financial relationships that could be construed as a potential conflict of interest.

## Footnotes

1 http://www.ncbi.nlm.nih.gov/genomes/static/Pipeline.html
