# Supplementary material for: Pectobacterium atrosepticum and Pectobacterium carotovorum Harbor Distinct, Independently Acquired Integrative and Conjugative Elements Encoding Coronafacic Acid that Enhance Virulence on Potato Stems
Source: Front Microbiol. 2016 Mar 31;7:397. doi: 10.3389/fmicb.2016.00397 (PMC4814525; doi:10.3389/fmicb.2016.00397)
Supplement: Supplementary file 1 [file Table_1.DOCX]

**Table S1.** Presence of loci associated with the *cfa* biosynthetic cluster and HAI2 in *Pectobacterium* infecting potato.

| Isolate Designation | | Isolate Origin | | | | *cfa* cluster | |  | Other HAI2 associated loci | | | | |
| --- | --- | --- | --- | --- | --- | --- | --- | --- | --- | --- | --- | --- | --- |
|  |  |  |  |  |  | *cfa6* | *cfa7* | *ECA0516* | *ECA0525* | *ECA0532* | *ECA0614* | *attL* | *attR* |
| ***Pectobacterium atrosepticum*** | | | | | |  | |  |  |  |  |  |  |
| SCRI1043 | United Kingdom^a^ | | | | **+** | **+** | **+** | **+** | **+** | **+** | **+** | **+** |  |
| ICMP 1526^T^ | United Kingdom^b^ | | | | **+** | **+** | **+** | **+** | **+** | **+** | **-** | **-** |  |
| ICMP 11525 | New Zealand^b^ | | | | **+** | **+** | **+** | **+** | **+** | **+** | **+** | **+** |  |
| ICMP 11299 | New Zealand^b^ | | | | **+** | **+** | **+** | **+** | **+** | **+** | **+** | **+** |  |
| ICMP 4398 | New Zealand^b^ | | | | **+** | **+** | **+** | **+** | **+** | **+** | **+** | **+** |  |
| ICMP 8975 | New Zealand^b^ | | | | **+** | **+** | **+** | **+** | **+** | **+** | **+** | **+** |  |
| NZEC13 | New Zealand | | | | **+** | **+** | **+** | **+** | **+** | **+** | **+** | **+** |  |
| NZEC16 | New Zealand | | | | **+** | **+** | **+** | **+** | **+** | **+** | **+** | **+** |  |
| NZEC21 | New Zealand | | | | **+** | **+** | **+** | **+** | **+** | **+** | **+** | **+** |  |
| NZEC22 | New Zealand | | | | **+** | **+** | **+** | **+** | **+** | **+** | **+** | **+** |  |
| ***Pectobacterium carotovorum* subsp*. brasiliensis*** | | | | | |  | |  |  |  |  |  |  |
| ICMP 19477 | New Zealand | | | | **+** | **+** | - | - | - | - | - | - |  |
| NZEC8 | New Zealand | | | | - | - | - | - | - | - | - | - |  |
| NZEC25 | New Zealand | | | | - | - | - | - | - | - | - | - |  |
| NZEC43 | New Zealand | | | | - | - | - | - | - | - | - | - |  |
| NZEC128 | New Zealand | | | | - | - | - | - | - | - | - | - |  |
| NZEC129 | New Zealand | | | | - | - | - | - | - | - | - | - |  |
| NZEC130 | New Zealand | | | | - | - | - | - | - | - | - | - |  |
| NZEC132 | New Zealand | | | | - | - | - | - | - | - | - | - |  |
| NZEC137 | New Zealand | | | | - | - | - | - | - | - | - | - |  |
| NZEC138 | New Zealand | | | | - | - | - | - | - | - | - | - |  |
| NZEC140 | New Zealand | | | | - | - | - | - | - | - | - | - |  |
| NZEC142 | New Zealand | | | | - | - | - | - | - | - | - | - |  |
| NZEC143 | New Zealand | | | | - | - | - | - | - | - | - | - |  |
| NZEC149 | New Zealand | | | | - | - | - | - | - | - | - | - |  |
| NZEC150 | New Zealand | | | | **+** | **+** | - | - | - | - | - | - |  |
| NZEC152 | New Zealand | | | | - | - | - | - | - | - | - | - |  |
| NZEC153 | New Zealand | | | | - | - | - | - | - | - | - | - |  |
| NZEC154 | New Zealand | | | | - | - | - | - | - | - | - | - |  |
| ***Pectobacterium carotovorum* subsp*. carotovorum*** | | | | | |  | |  |  |  |  |  |  |
| ICMP 5702^T^ | Denmark^b^ | | | | - | - | - | - | - | - | - | - |  |
| UGC25 | Peru^c^ | | | | **+** | **+** | NT | NT | NT | NT | NT | NT |  |
| UGC30 | Peru^c^ | | | | **+** | **+** | NT | NT | NT | NT | NT | NT |  |
| UGC31 | Peru^c^ | | | | **+** | **+** | NT | NT | NT | NT | NT | NT |  |
| UGC32 | Peru^c^ | | | | **+** | **+** | - | - | - | + | - | + |  |
| UGC33 | Peru^c^ | | | | **+** | **+** | NT | NT | NT | NT | NT | NT |  |
| UGC41 | United Kingdom^c^ | | | | - | - | - | - | - | - | - | - |  |
|  |  | | | |  |  |  |  |  |  |  |  |  |
|  |  | | | |  |  |  |  |  |  |  |  |  |
| Isolate Designation | Isolate Origin | | | | *cfa* cluster | | Other HAI2 associated loci | | | | | |  |
|  |  |  |  |  | *cfa6* | *cfa7* | *ECA0516* | *ECA0525* | *ECA0532* | *ECA0614* | *attL* | *attR* |  |
| NZEC5 | New Zealand | | | | - | - | - | - | - | - | - | - |  |
| NZEC6 | New Zealand | | | | - | - | - | - | - | - | - | - |  |
| NZEC7 | New Zealand | | | | - | - | - | - | - | - | - | - |  |
| NZEC14 | New Zealand | | | | - | - | - | - | - | - | - | - |  |
| NZEC19 | New Zealand | | | | - | - | - | - | - | - | - | - |  |
| NZEC20 | New Zealand | | | | - | - | - | - | - | - | - | - |  |
| NZEC23 | New Zealand | | | | - | - | - | - | - | - | - | - |  |
| NZEC24 | New Zealand | | | | - | - | - | - | - | - | - | - |  |
| NZEC31 | New Zealand | | | | - | - | - | - | - | - | - | - |  |
| NZEC32 | New Zealand | | | | - | - | - | - | - | - | - | - |  |
| NZEC38 | New Zealand | | | | - | - | - | - | - | - | - | - |  |
| NZEC68 | New Zealand | | | | - | - | - | - | - | - | - | - |  |
| NZEC78 | New Zealand | | | | - | - | - | - | - | - | - | - |  |
| NZEC89 | New Zealand | | | | - | - | - | - | - | - | - | - |  |
| NZEC90 | New Zealand | | | | - | - | - | - | - | - | - | - |  |
| NZEC91 | New Zealand | | | | - | - | - | - | - | - | - | - |  |
| NZEC93 | New Zealand | | | | - | - | - | - | - | - | - | - |  |
| NZEC110 | New Zealand | | | | - | - | - | - | - | - | - | - |  |
| NZEC115 | New Zealand | | | | - | - | - | - | - | - | - | - |  |
| NZEC118 | New Zealand | | | | - | - | - | - | - | - | - | - |  |
| NZEC119 | New Zealand | | | | - | - | - | - | - | - | - | - |  |
| NZEC121 | New Zealand | | | | - | - | - | - | - | - | - | - |  |
| NZEC124 | New Zealand | | | | - | - | - | - | - | - | - | - |  |
| NZEC126 | New Zealand | | | | - | - | - | - | - | - | - | - |  |
| NZEC131 | New Zealand | | | | - | - | - | - | - | - | - | - |  |
| NZEC133 | New Zealand | | | | - | - | - | - | - | - | - | - |  |
| NZEC134 | New Zealand | | | | - | - | - | - | - | - | - | - |  |
| NZEC139 | New Zealand | | | | - | - | - | - | - | - | - | - |  |
| NZEC141 | New Zealand | | | | - | - | - | - | - | - | - | - |  |
| NZEC144 | New Zealand | | | | - | - | - | - | - | - | - | - |  |
| NZEC146 | New Zealand | | | | - | - | - | - | - | - | - | - |  |
| NZEC147 | New Zealand | | | | - | - | - | - | - | - | - | - |  |
| NZEC155 | New Zealand | | | | - | - | - | - | - | - | - | - |  |
| NZEC157 | New Zealand | | | | - | - | - | - | - | - | - | - |  |
| NZEC158 | New Zealand | | | | - | - | - | - | - | - | - | - |  |
| NZEC161 | New Zealand | | | | - | - | - | - | - | - | - | - |  |
| NZEC164 | New Zealand | | | | - | - | - | - | - | - | - | - |  |
| NZEC165 | New Zealand | | | | - | - | - | - | - | - | - | - |  |
| NZEC166 | New Zealand | | | | - | - | - | - | - | - | - | - |  |
| NZEC168 | New Zealand | | | | - | - | - | - | - | - | - | - |  |
| NZEC169 | New Zealand | | | | - | - | - | - | - | - | - | - |  |
| NZEC170 | New Zealand | | | | - | - | - | - | - | - | - | - |  |
| NZEC172 | New Zealand | | | | - | - | - | - | - | - | - | - |  |
| NZEC173 | New Zealand | | | | - | - | - | - | - | - | - | - |  |
| Isolate Designation | | Isolate Origin | | | | *cfa* cluster | | Other HAI2 associated loci | | | | | |
|  |  |  |  |  |  | *cfa6* | *cfa7* | *ECA0516* | *ECA0525* | *ECA0532* | *ECA0614* | *attL* | *attR* |
| NZEC174 | New Zealand | | | | - | - | - | - | - | - | - | - |  |
| NZEC175 | New Zealand | | | | - | - | - | - | - | - | - | - |  |
| NZEC176 | New Zealand | | | | - | - | - | - | - | - | - | - |  |
| NZEC177 | New Zealand | | | | - | - | - | - | - | - | - | - |  |
| NZEC179 | New Zealand | | | | - | - | - | - | - | - | - | - |  |
| NZEC180 | New Zealand | | | | - | - | - | - | - | - | - | - |  |
| NZEC181 | New Zealand | | | | - | - | - | - | - | - | - | - |  |
| NZEC182 | New Zealand | | | | - | - | - | - | - | - | - | - |  |
| NZEC183 | New Zealand | | | | - | - | - | - | - | - | - | - |  |
| NZEC185 | New Zealand | | | | - | - | - | - | - | - | - | - |  |
| NZEC188 | New Zealand | | | | - | - | - | - | - | - | - | - |  |
| ***Pectobacterium wasabiae*** | | | |  | |  |  |  |  |  |  |  |  |
| ICMP 9121^T^ | Japan^b^ | | | | - | - | - | - | - | - | - | - |  |
| NZEC9 | New Zealand | | | | - | - | - | - | - | - | - | - |  |
| NZEC10 | New Zealand | | | | - | - | - | - | - | - | - | - |  |
| NZEC12 | New Zealand | | | | - | - | - | - | - | - | - | - |  |
| NZEC8974 | New Zealand | | | | - | - | - | - | - | - | - | - |  |
| ***Pectobacterium* sp.** | | |  | |  | |  |  |  |  |  |  |  |
| NZEC211 | New Zealand | | | | - | - | - | - | - | - | - | - |  |
| NZEC210 | New Zealand | | | | - | - | - | - | - | - | - | - |  |
| NZEC127 | New Zealand | | | | - | - | - | - | - | - | - | - |  |
| NZEC135 | New Zealand | | | | - | - | - | - | - | - | - | - |  |
| NZEC151 | New Zealand | | | | - | - | - | - | - | - | - | - |  |

+, amplification of PCR product; -, no amplicon generated; NT, not tested.

^T^ Type strain.

^≠^ Strain defined as an intermediate of *P. atrosepticum* and *P. carotovorum* subsp. *carotovorum* by phylogenetic analysis (Pitman *et al*., 2008).

^a^ I. Toth, Scottish Crop Research Institute, Invergowrie, Dundee, UK.

^b^ International Collection of Microbes from Plants, Landcare, Auckland, New Zealand.

^c^ Slawiak and Lojkowska (2009).
