# Supplementary material for: Pectobacterium atrosepticum and Pectobacterium carotovorum Harbor Distinct, Independently Acquired Integrative and Conjugative Elements Encoding Coronafacic Acid that Enhance Virulence on Potato Stems
Source: Front Microbiol. 2016 Mar 31;7:397. doi: 10.3389/fmicb.2016.00397 (PMC4814525; doi:10.3389/fmicb.2016.00397)
Supplement: Supplementary file 2 [file Table_2.DOCX]

**Table S2.** TBLASTX results for HAI2 in *Pectobacterium atrosepticum* SCRI1043 and the putative ICEs encoding CFA in *P. atrosepticum* ICMP1526, *P. carotovorum* subsp. *carotovorum* UGC32 and *P. carotovorum* subsp. *brasiliensis* ICMP19477.

| SCR1043 Gene ID and homologues in ICMP1526, UGC32 and ICMP19477 | | | | | | | SCRI1043 CDS Annotation | Strand | Length of SCRI1043 CDS (aa) | Predicted product | Blast search result | E value | Predicted Function |
| --- | --- | --- | --- | --- | --- | --- | --- | --- | --- | --- | --- | --- | --- |
| SCRI1043 | ICMP1526 | E value | UGC32^≠^ | E value | ICMP19477 | E value |  |  |  |  |  |  |  |
| ECA0516 | KCQ_12840 | 0.0 | GO33_02620 | 2e-170 | KCO_08800 | 7e-120 | *soj* | + | 888 | Plasmid partitioning related protein | Q8Z1M3 | 6.65e-117 | Plasmid replication |
| ECA0517 | KCQ_12845 | 1e-158 |  |  |  |  | Hypothetical | + | 690 | Unknown | B4EY09 | 5.8e-13 | Unknown |
| ECA0518 | KCQ_12850 | 4e-92 | *592297-592700* | 6e-22 | KCO_08795 | 3e-31 | Hypothetical | + | 393 | Unknown | No hits |  | Unknown |
| ECA0519 | KCQ_12855 | 0.0 | GO33_02630 | 0.0 | KCO_08790 | 9e-138 | DNA helicase | + | 1395 | DnaB-like DNA helicase | B4EY08 | 1.38e-126 | Plasmid replication |
| ECA0520 | KCQ_12860 | 0.0 | GO33_02635 | 0.0 | KCO_08785 | 9e-160 | Hypothetical | + | 1725 | Unknown | A8G8H3 | 6.41e-124 | Unknown |
| ECA0521 | KCQ_12865 | 7e-60 | GO33_02640 | 3e-133 |  |  | Hypothetical | + | 273 | Unknown (Doubtful CDS) | No hits |  | Unknown |
| ECA0522 | KCQ_12870 | 8e-141 | GO33_02645 | 2e-91 | KCO_08770 | 3e-66 | Hypothetical | + | 615 | Unknown | A8GJB7 | 4.1e-67 | Unknown |
| ECA0522A | KCQ_12875 | 1e-58 | *596696-596950* | 96-108 | KCO_08765 | 2e-15 | Hypothetical | + | 255 | Unknown | A8G8H6 | 2.35e-18 | Unknown |
| ECA0523 | KCQ_12885 | 0.0 | GO33_02650 | 0.0 | KCO_08750 | 6e-115 | Hypothetical | + | 1197 | Unknown | Q7N7S3 | 5.18e-116 | Unknown |
| ECA0524 | KCQ_12890 | 0.0 | GO33_02655 | 1e-79 | KCO_08745 | 4e-57 | Hypothetical | + | 798 | Unknown | A8G8H8 | 4.97e-65 | Unknown |
| ECA0525 | KCQ_12895 | 0.0 | GO33_02660 | 2e-170 | KCO_08740 | 0.0 | *topB* | + | 2019 | Topoisomerase III | A8GJB2 | 0.0 | Plasmid replication |
| ECA0526 | KCQ_12896 | 3e-39 | GO33_02665 | 1e-71 |  |  | Hypothetical | - | 177 | Putative phage protein | Q94MN5 | 2.3 | Unknown |
| ECA0527 |  |  | *601998-602108* | 3e-23 |  |  | Hypothetical | - | 156 | Unknown (Doubtful CDS) | No hits |  | Unknown |
| ECA0528 | KCQ_12900 | 4e-108 | GO33_02670 | 8e-87 |  |  | Hypothetical | + | 492 | Unknown | A8G8I1 | 6.62e-54 | Unknown |
| ECA0531 | KCQ_12910 | 8e-100 | GO33_02685 | 1e-146 | KCO_08720 | 1e-68 | Hypothetical | + | 438 | Plasmid-related protein | Q7N4J8 | 9.76e-57 | Unknown |
| ECA0532 | KCQ_12920 | 0.0 | GO33_02690 | 4e-58 | KCO_08705 | 1e-17 | *pilL* | + | 1317 | Type IV pilus protein | Q6X921 | 8.34e-78 | Conjugation |
| ECA0533 | KCQ_12925 | 3e-94 | GO33_02695 | 0.0 |  |  | *pilM* | + | 447 | Type IV pilus protein | O07371 | 2.48e-05 | Conjugation |
| ECA0534 | KCQ_12930 | 0.0 | GO33_02700 | 0.0 | KCO_08695 | 5e-76 | *pilN* | + | 1665 | Type IV pilus protein | Q79VR6 | 9.87e-90 | Conjugation |
| ECA0535 | KCQ_12935 | 0.0 |  |  |  |  | *pilO* |  | 1303 | Type IV pilus protein | Q7N7Q6 | 2e-23 | Conjugation |
| ECA0537 | KCQ_12940 | 0.0 | GO33_02710 | 0.0 |  |  | *pilP* | + | 498 | Type IV pilus protein | Q9RHF2 | 0.21 | Conjugation |
| ECA0538 | KCQ_12945 | 0.0 | GO33_02715 | 0.0 | KCO_08680 | 2e-76 | *pilQ* | + | 1560 | Type IV pilus protein | A9N2V2 | 2.50e-71 | Conjugation |
| ECA0539 | 978512-978664 | 2e-75 | *611057-611209* | 5e-79 |  |  |  | + | 153 | Unknown (Doubtful CDS) | No hits |  | Unknown |
| ECA0540 | KCQ_12950 | 0.0 | GO33_02720 | 0.0 | KCO_08675 | 5e-36 | *pilR* | + | 1038 | Type IV pilus protein | Q79VS0 | 1.11e-31 | Conjugation |
| ECA0541 | KCQ_12955 | 1e-149 | GO33_02725 | 7e-106 |  |  | *pilS* | + | 606 | Type IV pilus protein | Q8KR25 | 1.20e-05 | Conjugation |
| ECA0542 | KCQ_12960 | 7e-122 | GO33_02730 | 5e-102 | KCO_08665 | 5e-53 | *pilT* | + | 519 | Type IV pilus protein | Q7BLG8 | 3.20e-36 | Conjugation |
| ECA0543 | KCQ_12965 | 2e-145 | GO33_02735 | 5e-113 |  |  | *pilU* | + | 660 | Type IV pilus protein | Q9R2J4 | 0.029 | Conjugation |
| ECA0544 | KCQ_12970 | 0.0 | GO33_02740 | 0.0 | KCO_08655 | 1e-15 | *pilV* | + | 1395 | Type IV pilus protein | Q79JW3 | 7.57e-32 | Conjugation |
| ECA0545 | KCQ_12860 | 0.049 | *616223-615745* | 0.0 |  |  | C-terminal for *pilV* | + | 480 | Type IV pilus protein | B0HU65 | 2.64e-25 | Conjugation |
| ECA0545A | KCQ_12080 | 0.39 | *615513-615726* | 1e-105 |  |  | C-terminal for *pilV* | + | 216 | Type IV pilus protein |  |  | Conjugation |
| ECA0546 | KCQ_12975 | 0.0 | GO33_02750 | 0.0 |  |  | *rci* | + | 1128 | Shufflon-specific DNA recombinase | Q7BLG6 | 5.63e-133 | Conjugation |
| ECA0547 | KCQ_12976 | 2e-112 | *618062-618448* | 0.0 |  |  | *pilK* | + | 492 | Putative exported protein | Q8Z1K9 | 1.70e-25 | Unknown |
| ECA0548 | KCQ_12980 | 6e-174 | GO33_02755 | 0.0 |  |  | *traE* | + | 801 | Transferase (TraE) | Q8Z1K8 | 3.38e-37 | Conjugation |
| ECA0549 | KCQ_12985 | 0.0 | GO33_02760 | 0.0 |  |  | *traF* | + | 1203 | Transferase (TraF) | B7N004 | 1.43e-105 | Conjugation |
| ECA0550 | KCQ_12990 | 4e-104 |  |  |  |  | Hypothetical | + | 450 | Plasmid protein | Q8KJV9 | 4.46e-33 | Unknown |
| ECA0551 | KCQ_12995 | 1e-67 |  |  |  |  | Hypothetical | + | 300 | Putative membrane protein | BAC14027 | 1.4 | Unknown |
| ECA0552 | KCQ_13000 | 2e-161 | GO33_02765 | 1e-107 |  |  | Hypothetical | + | 693 | Putative membrane protein | B1JIN8 | 5.02e-57 | Unknown |
| ECA0553 | KCQ_13005 | 6e-88 | *622751-622830* | 5e-12 |  |  | Hypothetical | + | 375 | Putative exported protein | No hits |  | Unknown |
| ECA0554 | KCQ_13010 | 0.0 | *622933-623807* | 0.0 |  |  | Hypothetical | + | 876 | Putative membrane protein | Q8Z1K7 | 1.27e-27 | Unknown |
| ECA0555 | KCQ_13015 | 2e-166 | GO33_02780 | 5e-141 | KCO_08645 | 4e-89 | Hypothetical | + | 714 | Putative exported protein | A8G8J2 | 5.23e-81 | Unknown |
| ECA0556 | KCQ_13020 | 7e-137 | GO33_02785 | 1e-100 | KCO_08640 | 3e-64 | Hypothetical | + | 588 | Lytic transglycosylase | A8GJI6 | 3.86e-86 | Unknown |
| ECA0557 | KCQ_13025 | 1e-123 | GO33_02790 | 3e-94 | KCO_08635 | 2e-39 | Hypothetical | + | 522 | Putative exported protein | A8GJI5 | 3.97e-42 | Unknown |
| ECA0558 | KCQ_13030 | 2e-140 | GO33_02795 | 1e-114 |  |  | Hypothetical | + | 618 | Restriction endonuclease | A8GJA2 | 1.53e-53 | DNA cleavage |
| ECA0559 | KCQ_13031 | 1e-107 | GO33_02800 | 0.0 | KCO_08630 | 1e-14 | Hypothetical | + | 483 | Putative membrane protein | No hits |  | Unknown |
| ECA0560 | KCQ_13035 | 0.0 | GO33_02805 | 0.0 | KCO_08625 | 0.0 | Hypothetical | + | 2097 | Plasmid transfer protein | B7MZR2 | 0.0 | Plasmid transfer |
| ECA0561 | KCQ_13040 | 0.0 | GO33_02810 | 5e-115 | KCO_08620 | 4e-69 | Hypothetical | + | 756 | Putative membrane protein | A8GJI2 | 5.44e-79 | Unknown |
| ECA0562 | KCQ_13045 | 1e-42 | GO33_02815 | 9e-33 |  |  | Hypothetical | + | 195 | Unknown | No hits |  | Unknown |
| ECA0563 | KCQ_13050 | 6e-115 |  |  |  |  | Hypothetical | - | 540 | Unknown | A8G8J8 | 7.56e-34 | Unknown |
| ECA0564 | KCQ_13055 | 2e-74 | GO33_02830 | 9e-48 | KCO_08610 | 7e-23 | Hypothetical | + | 321 | Plasmid protein | A8GJI1 | 6.06e-30 | Unknown |
| ECA0565 | KCQ_13060 | 4e-52 | GO33_02835 | 4e-30 |  |  | Hypothetical | + | 240 | Putative membrane protein | Q6GUD5 | 1.55e-17 | Unknown |
| ECA0566 | KCQ_13065 | 1e-76 | GO33_02840 | 1e-47 | KCO_08600 | 9e-20 | Hypothetical | + | 342 | Putative membrane protein | Q6GUD6 | 1.83e-26 | Unknown |
| ECA0567 | KCQ_13070 | 2e-81 | GO33_02845 | 5e-46 | KCO_08595 | 2e-20 | Hypothetical | + | 363 | Putative membrane protein | A8GJH8 | 4.09e-26 | Unknown |
| ECA0568 | KCQ_13075 | 3e-148 | GO33_02850 | 2e-124 | KCO_08590 | 2e-102 | Hypothetical | + | 657 | Putative membrane protein | Q6GUD9 | 4.71e-91 | Unknown |
| ECA0569 | KCQ_13080 | 0.0 | GO33_02855 | 1e-147 | KCO_08585 | 1e-90 | Hypothetical | + | 927 | Putative membrane protein | A8GJH6 | 2.17e-97 | Unknown |
| ECA0570 | KCQ_13085 | 0.0 | GO33_02860 | 0.0 | KCO_08580 | 2e-144 | Hypothetical | + | 1533 | Putative exported protein | B7N358 | 2.00e-162 | Unknown |
| ECA0571 | KCQ_13090 | 1e-70 | GO33_02865 | 5e-164 |  |  | Hypothetical | + | 336 | Unknown | No hits |  | Unknown |
| ECA0572 | KCQ_13095 | 2e-87 | GO33_02870 | 5e-79 | KCO_08565 | 1e-49 | Hypothetical | + | 411 | Putative lipoprotein | A8G8K6 | 2.00e-49 | Unknown |
| ECA0573 | KCQ_13100 | 0.0 | GO33_02875 | 0.0 | KCO_08560 | 0.0 | Hypothetical | + | 2862 | Plasmid transfer protein | A8G8K7 | 0.0 | Conjugation |
| ECA0574 | KCQ_13105 | 9e-88 | *639495-639940* | 1e-179 | KCO_08555 | 8e-16 | Hypothetical | + | 447 | Unknown | A8G8K8 | 6.93e-26 | Unknown |
| ECA0575 | KCQ_13106 | 2e-96 |  |  |  |  | Hypothetical | - | 435 | Putative membrane protein | Q03860 | 0.59 | Unknown |
| ECA0576 | KCQ_13110 | 5e-94 | GO33_02890 | 2e-124 | KCO_08520 | 2e-30 | Hypothetical | + | 405 | Putative exported protein | A8G8L8 | 1.19e-30 | Unknown |
| ECA0577 | KCQ_13115 | 0.0 | GO33_02895 | 0.0 | KCO_08515 | 6e-98 | Hypothetical | + | 975 | Putative exported protein | A8G8L9 | 7.17e-139 | Unknown |
| ECA0578 | KCQ_13120 | 0.0 | *642718-644142* | 0.0 | KCO_08510 | 8e-178 | Hypothetical | + | 1425 | Putative exported protein | A8G8M0 | 0.0 | Unknown |
| ECA0579 | KCQ_13125 | 5e-88 | GO33_02905 | 2e-49 |  |  | Hypothetical | + | 372 | Putative exported protein | A8GJ77 | 8.99e-26 | Unknown |
| ECA0580 | KCQ_13130 | 0.0 | GO33_02910 | 0.0 | KCO_08500 | 6e-122 | Hypothetical | + | 1533 | Putative membrane protein | A8GJ76 | 0.0 | Unknown |
| ECA0581 | KCQ_13135 | 8e-88 | GO33_02915 | 8e-80 |  |  | Hypothetical | - | 390 | Putative membrane protein | A8G8M3 | 1.27e-11 | Unknown |
| ECA0582 | KCQ_13140 | 4e-86 |  |  |  |  | Hypothetical | - | 360 | Plasmid stable inheritance protein | B0QUG5 | 1.67e-24 | Growth inhibitor |
| ECA0583 | KCQ_13145 | 2e-73 |  |  |  |  | Hypothetical | - | 303 | Plasmid-related protein | O52204 | 1.7 | Transcriptional regulator |
| ECA0584 | KCQ_13150 | 0.0 | GO33_02930 | 0.0 |  |  | Hypothetical | + | 1872 | Restriction enzyme α | B7N031 | 0.0 | Unknown |
| ECA0585 | KCQ_13155 | 0.0 | *651171-652066* | 0.0 |  |  | Hypothetical | + | 987 | Restriction enzyme β | B7N030 | 1.07e-97 | Unknown |
| ECA0586 | KCQ_13156 | 1e-29 | *652199-652325* | 5e-45 |  |  | Hypothetical | + | 141 | Unknown | No hits |  | Unknown |
| ECA0586A | KCQ_13157 | 6e-35 | *652390-652556* | 3e-55 |  |  | Hypothetical | + | 168 | Unknown | O27832 | 2.1 | Unknown |
| ECA0587 | KCQ_13160 | 0.0 | GO33_02945 | 1e-161 | KCO_08485 | 2e-161 | Hypothetical | + | 891 | Putative integrase | A9N3W8 | 2.38e-114 | DNA recombination |
| ECA0588 | KCQ_13165 | 4e-64 | *654070-654360* | 2e-117 |  |  | Hypothetical | - | 291 | Plasmid-related protein | A4IUN6 | 3.60e-14 | Unknown |
| ECA0589 | KCQ_13170 | 8e-71 | GO33_02950 | 4e-42 |  |  | Hypothetical | + | 312 | Unknown | No hits |  | Unknown |
| ECA0590 | KCQ_13175 | 3e-72 | GO33_02955 | 3e-53 |  |  | Hypothetical | + | 357 | Unknown | No hits |  | Unknown |
| ECA0591 | KCQ_13180 | 4e-109 | GO33_02960 | 5e-63 | KCO_08475 | 1e-98 | Hypothetical | + | 474 | Unknown | No hits |  | Unknown |
| ECA0592 | KCQ_13185 | 1e-96 | GO33_02965 | 1e-78 | KCO_08460 | 6e-73 | Hypothetical | + | 405 | Unknown | No hits |  | Unknown |
| ECA0593 | KCQ_13190 | 5e-94 | GO33_02970 | 9e-70 | KCO_08455 | 2e-70 | Hypothetical | + | 414 | Putative DNA repair protein | B8E5D0 | 4.45e-33 | Unknown |
| ECA0594 | KCQ_13200 | 9e-135 | GO33_02975, 80 | 1e-86 | KCO_08450 | 9e-116 | Hypothetical | + | 597 | Unknown | Q7N7J4 | 9.04e-24 | Unknown |
| ECA0595 | KCQ_13205 | 2e-58 | GO33_02985 | 3e-30 | KCO_08440 | 4e-51 | Hypothetical | + | 273 | Unknown | B6VNI8 | 1.60e-14 | Unknown |
| ECA0596 | KCQ_13210 | 5e-110 | GO33_02990 | 2e-82 | KCO_08435 | 6e-97 | Hypothetical | + | 486 | Unknown | Q8Z1H5 | 2.11e-41 | Unknown |
| ECA0597 | KCQ_13215 | 0.0 | GO33_02995 | 0.0 | KCO_08430 | 0.0 | Hypothetical | + | 1962 | Unknown | Q8Z1H4 | 1.15-148 | Unknown |
| ECA0598 | KCQ_13220 | 3e-174 | GO33_03000 | 2e-151 | KCO_08425 | 4e-169 | Hypothetical | + | 894 | Unknown | Q8Z1H3 | 1.81e-45 | Unknown |
| ECA0599 | KCQ_13225 | 7e-179 | GO33_03005 | 8e-173 | KCO_08420 | 1e-178 | Hypothetical | + | 921 | Unknown | Q8Z1H2 | 1.50e-50 | Unknown |
| ECA0600 | KCQ_13230 | 0.0 | GO33_03015 | 0.0 | KCO_08415 | 0.0 | *cfa8B* | - | 1314 | Putative oxidoreductase | O69071 | 5.42e-170 | Cfa production |
| ECA0601 | KCQ_13235 | 6e-96 | GO33_03020 | 2e-77 | KCO_08410 | 4e-95 | *cfa8A* | - | 432 | Putative oxidoreductase | Q87W68 | 1.26e-27 | Cfa production |
| ECA0602 | KCQ_13240 | 0.0 | GO33_03025 | 0.0 | KCO_08405 | 0.0 | *cfa7* | - | 6387 | Polyketide synthase | Q87W69 | 0.0 | Cfa production |
| ECA0603 | KCQ_13245 | 0.0 | GO33_03030 | 0.0 | KCO_08400 | 0.0 | *cfa6* | - | 8142 | Polyketide synthase | Q87W70 | 0.0 | Cfa production |
| ECA0604 | KCQ_13250 | 0.0 | GO33_03035 | 0.0 | KCO_08395 | 0.0 | *cfa5* | - | 1440 | Coronafacic acid synthase | Q87W71 | 4.17e-110 | Cfa production |
| ECA0605 | KCQ_13255 | 3e-127 | GO33_03040 | 5e-24 | KCO_08390 | 2e-120 | *cfa4* | - | 534 | Coronafacic acid synthase | Q87W72 | 5.06e-35 | Cfa production |
| ECA0606 | KCQ_13260 | 0.0 | GO33_03045 | 0.0 | KCO_08385 | 0.0 | *cfa3* | - | 1146 | Cfa-β-ketoacylsynthase | Q87W73 | 1.99e-117 | Cfa production |
| ECA0607 | KCQ_13265 | 2e-116 | GO33_03050 | 1e-93 | KCO_08380 | 1e-106 | *cfa2* | - | 498 | Coronafacic acid dehydratase | Q87W74 | 3.11e-67 | Cfa production |
| ECA0608 | KCQ_13270 | 3e-60 | GO33_03055 | 1e-37 | KCO_08375 | 4e-52 | *cfa1* | - | 276 | Cfa-acyl carrier protein | Q87W75 | 3.78-16 | Cfa production |
| ECA0609 | KCQ_13275 | 0.0 | GO33_03060 | 0.0 | KCO_08370 | 0.0 | *cfl* | - | 1563 | Coronafacate ligase | Q87W76 | 1.78e-101 | Cfa production |
| ECA0610 | KCQ_13280 | 0.0 | GO33_03065 | 0.0 | KCO_08365 | 0.0 | *lysR*-like regulator | + | 963 | Transcriptional regulator | Q39NJ9 | 9.56e-35 | Transcriptional regulation |
| ECA0611 | KCQ_13285 | 0.0 | GO33_03070 | 0.0 | KCO_08360 | 0.0 | DNA helicase | + | 1461 | REP-family DNA helicase | A8GJ55 | 1.44e-174 | DNA replication and repair |
| ECA0612 | KCQ_13290 | 7e-114 | GO33_03075 | 2e-96 |  |  | Hypothetical | + | 492 | DinB-family protein | A8G8N0 | 3.17e-64 | SOS-like regulation |
| ECA0613 | KCQ_13295 | 0.0 | GO33_03080 | 0.0 | KCO_08345 | 0.0 | Hypothetical | + | 1599 | Conjugative relaxase TraI_2 | A7ZRD0 | 8.80e-96 | Conjugation |
| ECA0614 | KCQ_13300 | 0.0 | GO33_03085 | 0.0 | KCO_08340 | 0.0 | *xerC* | + | 1023 | Phage integrase similar to XerC | A8GJ53 | 2.61e-102 | DNA recombination |

*frameshift mutation

^≠^ italized values represent the genome coordinates in UGC32 for homologues of HAI2 CDSs identified manually that were not defined as CDSs by annotation using the PGAAP pipeline.
