# Supplementary material for: Pectobacterium atrosepticum and Pectobacterium carotovorum Harbor Distinct, Independently Acquired Integrative and Conjugative Elements Encoding Coronafacic Acid that Enhance Virulence on Potato Stems
Source: Front Microbiol. 2016 Mar 31;7:397. doi: 10.3389/fmicb.2016.00397 (PMC4814525; doi:10.3389/fmicb.2016.00397)
Supplement: Supplementary file 3 [file Table_3.DOCX]

**Table S3.** The predicted function of proteins encoded by the coding domain sequences (CDSs) of PbN1_GI15 in *P. carotovorum* subsp. *brasiliensis* ICMP19477, as determined by the COG (Clusters of Orthologous Group) database.

| ICMP19477 locus tag | CDS Length (nt) | Strand | ICMP19477 CDS annotation | COG assignment | COG predicted product and function |
| --- | --- | --- | --- | --- | --- |
| KCO_08800 | 888 | + | hypothetical protein | COG1192 | ATPases involved in chromosome partitioning |
| KCO_08795 | 387 | + | hypothetical protein |  | Unknown |
| KCO_08790 | 1356 | + | replicative DNA helicase | COG0305 | Replicative DNA helicase |
| KCO_08785 | 1719 | + | hypothetical protein |  | Unknown |
| KCO_08780 | 240 | + | transcriptional regulator, TraR/DksA family protein | COG1734 | DnaK suppressor protein |
| KCO_08775 | 117 | + | hypothetical protein |  | Unknown |
| KCO_08770 | 582 | + | hypothetical protein |  | Unknown |
| KCO_08765 | 231 | + | hypothetical protein |  | Unknown |
| KCO_08760 | 198 | + | hypothetical protein |  | Unknown |
| KCO_08755 | 243 | + | hypothetical protein |  | Unknown |
| KCO_08750 | 1296 | + | hypothetical protein |  | Unknown |
| KCO_08745 | 753 | + | hypothetical protein |  | Unknown |
| KCO_08740 | 2013 | + | DNA topoisomerase III | COG0550 | Topoisomerase IA |
| KCO_08735 | 504 | + | hypothetical protein |  | Unknown |
| KCO_08730 | 522 | + | single-stranded DNA-binding protein | COG0629 | Single-stranded DNA-binding protein |
| KCO_08725 | 1881 | + | RNA-directed DNA polymerase | COG3344 | Retron-type reverse transcriptase |
| KCO_08720 | 315 | + | hypothetical protein |  | Unknown |
| KCO_08715 | 195 | - | hypothetical protein |  | Unknown |
| KCO_08710 | 117 | - | hypothetical protein |  | Unknown |
| KCO_08705 | 204 | + | pilus biogenesis protein PilL |  | Unknown |
| KCO_08700 | 438 | + | PilM protein |  | Unknown |
| KCO_08695 | 1722 | + | putative type IV pilus operon lipoprotein | COG1450 | Type II secretory pathway, component PulD |
| KCO_08690 | 1281 | + | pilin accessory protein PilO |  |  |
| KCO_08685 | 495 | + | putative type IV pilus protein | COG0469 | Pyruvate kinase |
| KCO_08680 | 1557 | + | putative type IV pilus protein | COG2804 | Unknown |
| KCO_08675 | 1089 | + | type IV pilus integral membrane protein | COG1459 | Type II secretory pathway, component PulF |
| KCO_08670 | 585 | + | putative type IV prepilin |  | Unknown |
| KCO_08665 | 480 | + | PilT protein | COG0741 | Soluble lytic murein transglycosylase and related regulatory proteins |
| KCO_08660 | 654 | + | type IV prepilin peptidase | COG1989 | Type II secretory pathway, prepilin signal peptidase PulO and related peptidases |
| KCO_08655 | 1482 | + | putative type IV pilus protein |  | Unknown |
| KCO_08650 | 627 | + | hypothetical protein |  | Unknown |
| KCO_08645 | 708 | + | hypothetical protein |  | Unknown |
| KCO_08640 | 651 | + | lytic transglycosylase catalytic | COG0741 | Soluble lytic murein transglycosylase and related regulatory proteins |
| KCO_08635 | 552 | + | hypothetical protein | COG0751 | Glycyl-tRNA synthetase, beta subunit |
| KCO_08630 | 384 | + | hypothetical protein |  | Unknown |
| KCO_08625 | 2112 | + | TraG-family protein | COG1053 | Succinate dehydrogenase/fumarate reductase,flavoprotein subunit |
| KCO_08620 | 753 | + | inner membrane protein |  | Unknown |
| KCO_08615 | 384 | + | hypothetical protein |  | Unknown |
| KCO_08610 | 315 | + | hypothetical protein |  | Unknown |
| KCO_08605 | 237 | + | hypothetical protein |  | Unknown |
| KCO_08600 | 345 | + | hypothetical protein |  | Unknown |
| KCO_08595 | 375 | + | hypothetical protein |  | Unknown |
| KCO_08590 | 660 | + | hypothetical protein | COG3162 | Predicted membrane protein |
| KCO_08585 | 828 | + | hypothetical protein |  | Unknown |
| KCO_08580 | 1542 | + | hypothetical protein |  | Unknown |
| KCO_08575 | 804 | + | hypothetical protein |  | Unknown |
| KCO_08570 | 462 | + | hypothetical protein |  | Unknown |
| KCO_08565 | 411 | + | hypothetical protein |  | Unknown |
| KCO_08560 | 2826 | + | hypothetical protein | COG3451 | Type IV secretory pathway |
| KCO_08555 | 384 | + | hypothetical protein |  | Unknown |
| KCO_08550 | 903 | + | hypothetical protein |  | Unknown |
| KCO_08545 | 228 | - | hypothetical protein |  | Unknown |
| KCO_08540 | 2382 | + | hypothetical protein |  | Unknown |
| KCO_08535 | 282 | + | hypothetical protein |  | Unknown |
| KCO_08530 | 894 | + | SMF protein | COG0758 | Predicted Rossmann fold nucleotide-binding protein involved in DNA uptake |
| KCO_08525 | 651 | + | hypothetical protein | COG1040 | Predicted amidophosphoribosyltransferases |
| KCO_08520 | 408 | + | hypothetical protein |  | Unknown |
| KCO_08515 | 768 | + | hypothetical protein |  | Unknown |
| KCO_08510 | 1428 | + | hypothetical protein |  | Unknown |
| KCO_08505 | 345 | + | hypothetical protein |  | Unknown |
| KCO_08500 | 1566 | + | hypothetical protein |  | Unknown |
| KCO_08495 | 723 | + | hypothetical protein |  | Unknown |
| KCO_08490 | 849 | - | hypothetical protein |  | Unknown |
| KCO_08485 | 888 | + | hypothetical protein | COG0582 | Integrase |
| KCO_08480 | 348 | + | hypothetical protein |  | Unknown |
| KCO_08475 | 474 | + | hypothetical protein |  | Unknown |
| KCO_08470 | 642 | + | hypothetical protein |  | Unknown |
| KCO_08465 | 195 | + | hypothetical protein |  | Unknown |
| KCO_08460 | 405 | + | hypothetical protein |  | Unknown |
| KCO_08455 | 414 | + | putative DNA repair protein | COG2003 | DNA repair proteins |
| KCO_08450 | 597 | + | hypothetical protein |  | Unknown |
| KCO_08445 | 438 | - | hypothetical protein |  | Unknown |
| KCO_08440 | 273 | + | hypothetical protein |  | Unknown |
| KCO_08435 | 486 | + | hypothetical protein |  | Unknown |
| KCO_08430 | 1941 | + | hypothetical protein | COG4227 | Anti-restriction protein |
| KCO_08425 | 894 | + | hypothetical protein |  | Unknown |
| KCO_08420 | 927 | + | hypothetical protein |  | Unknown |
| KCO_08415 | 1314 | - | Cfa8B | COG0604 | Putative oxidoreductase |
| KCO_08410 | 432 | - | Cfa8A | COG2050 | Putative oxidoreductase |
| KCO_08405 | 6387 | - | Cfa7 | COG3321 | Polyketide synthase modules and related proteins |
| KCO_08400 | 8130 | - | Cfa6 | COG3321 | Polyketide synthase modules and related proteins |
| KCO_08395 | 1443 | - | Cfa5 | COG0318 | Acyl-CoA synthetases (AMP-forming)/AMP-acidligases II |
| KCO_08390 | 534 | - | Cfa4 |  | Coronafacic acid synthetase component |
| KCO_08385 | 1146 | - | Cfa3 | COG0304 | 3-oxoacyl-(acyl-carrier-protein) synthase |
| KCO_08380 | 498 | - | Cfa2 | COG0764 | 3-hydroxymyristoyl/3-hydroxydecanoyl-(acyl carrier protein) dehydratases |
| KCO_08375 | 276 | - | Cfa1 | COG0236 | Acyl carrier protein |
| KCO_08370 | 1562 | - | Cfl | COG0318 | Coronafacate ligase |
| KCO_08365 | 963 | + | LysR family transcriptional regulator | COG0583 | Transcriptional regulator |
| KCO_08360 | 1464 | + | hypothetical protein | COG0210 | Superfamily I DNA and RNA helicases |
| KCO_08355 | 1107 | - | putative DNA-binding protein | COG1396 | Predicted transcriptional regulators |
| KCO_08350 | 672 | - | hypothetical protein |  | Unknown |
| KCO_08345 | 1599 | + | hypothetical protein |  | Unknown |
| KCO_08340 | 1023 | + | putative phage integrase | COG0582 | Integrase |
