# Supplementary material for: Pectobacterium atrosepticum and Pectobacterium carotovorum Harbor Distinct, Independently Acquired Integrative and Conjugative Elements Encoding Coronafacic Acid that Enhance Virulence on Potato Stems
Source: Front Microbiol. 2016 Mar 31;7:397. doi: 10.3389/fmicb.2016.00397 (PMC4814525; doi:10.3389/fmicb.2016.00397)

Figure S1. Comparative genomics of *P. atrosepticum* SCRI1043 and SCRI1043ΔHAI2 demonstrated that CRISPR-Cas-mediated genome editing had resulted in deletion of HAI2 in its entirety, but no other large scale deletions from the genome. The two genomes are shown as circular elements, with the genome coordinates provided in kilobases (kbp).

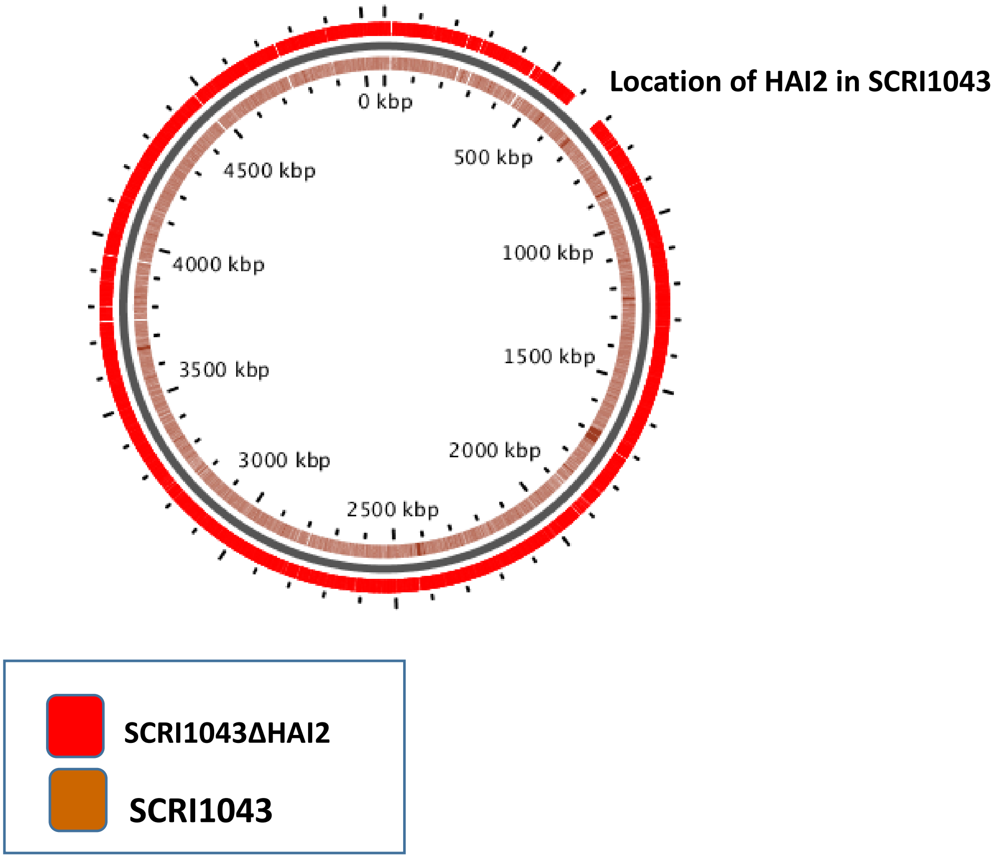

Supplement: Supplementary file 4 [file Image_1.PDF]
